# Influence of Corticospinal Tracts from Higher Order Motor Cortices on Recruitment Curve Properties in Stroke

**DOI:** 10.3389/fnins.2016.00079

**Published:** 2016-03-08

**Authors:** Kelsey A. Potter-Baker, Nicole M. Varnerin, David A. Cunningham, Sarah M. Roelle, Vishwanath Sankarasubramanian, Corin E. Bonnett, Andre G. Machado, Adriana B. Conforto, Ken Sakaie, Ela B. Plow

**Affiliations:** ^1^Department of Biomedical Engineering, Lerner Research Institute, Cleveland Clinic FoundationCleveland, OH, USA; ^2^School of Biomedical Sciences, Department of Neuroscience, Kent State UniversityKent, OH, USA; ^3^Center for Neurological Restoration, Neurosurgery, Neurological Institute, Cleveland Clinic FoundationCleveland, OH, USA; ^4^Neurology Clinical Division, Neurology Department, Clinics Hospital, São Paulo UniversitySão Paulo, Brazil; ^5^Hospital Israelita Albert EinsteinSão Paulo, Brazil; ^6^Department of Diagnostic Radiology, Imaging Institute, Cleveland Clinic FoundationCleveland, OH, USA; ^7^Department of Physical Medicine and Rehabilitation, Neurological Institute, Cleveland Clinic FoundationCleveland, OH, USA

**Keywords:** stimulus-response curve, recruitment curve, TMS, DTI, stroke

## Abstract

**Background:** Recruitment curves (RCs) acquired using transcranial magnetic stimulation are commonly used in stroke to study physiologic functioning of corticospinal tracts (CST) from M1. However, it is unclear whether CSTs from higher motor cortices contribute as well.

**Objective:** To explore whether integrity of CST from higher motor areas, besides M1, relates to CST functioning captured using RCs.

**Methods:** RCs were acquired for a paretic hand muscle in patients with chronic stroke. Metrics describing gain and overall output of CST were collected. CST integrity was defined by diffusion tensor imaging. For CST emerging from M1 and higher motor areas, integrity (fractional anisotropy) was evaluated in the region of the posterior limb of the internal capsule, the length of CST and in the region of the stroke lesion.

**Results:** We found that output and gain of RC was related to integrity along the length of CST emerging from higher motor cortices but not the M1.

**Conclusions:** Our results suggest that RC parameters in chronic stroke infer function primarily of CST descending from the higher motor areas but not M1. RCs may thus serve as a simple, in-expensive means to assess re-mapping of alternate areas that is generally studied with resource-intensive neuroimaging in stroke.

## Introduction

Transcranial magnetic stimulation (TMS) is a popular non-invasive technique to assess physiology of corticospinal tracts (CST; Di Lazzaro, 2004). TMS is able to gauge such physiology based on the principle of electromagnetic induction. Specifically, rapidly alternating currents form the basis for TMS. These are created by discharging a large capacitor into an insulated coiled wire. The produced currents then generate magnetic fields over the scalp and skull. Electrical currents are induced, which pass unimpeded to excite superficial areas like the primary motor cortices (M1). In M1, induced currents trigger volleys along descending CST pathways that produce motor evoked potentials (MEP) in contralateral muscles (Di Lazzaro, 2004). The resultant MEP amplitude is believed to reflect output of the CST pathways. With increasing TMS intensities, MEP amplitudes typically increase. By applying a range of increasing intensities, one can study incremental gains in MEPs that are plotted commonly as a stimulus-response or a recruitment curve. The slope of the curve and sum of MEP amplitudes signify gain and output of the descending CST (Devanne et al., 1997; Ridding and Rothwell, 1997; Boroojerdi et al., 2001; Monti et al., 2001; Ward et al., 2006).

TMS techniques are particularly relevant in stroke. This is because TMS can index function and recovery of the paretic upper limb by evaluating CST damage that is typical of stroke affecting the territory of the middle cerebral artery (Bogousslavsky and Regli, 1990; Johansen-Berg et al., 2002; Buffon et al., 2005). For example, several groups have established that the mere presence or absence of MEPs in paretic muscles can inform about clinical function (Ward et al., 2006; Stinear et al., 2007, 2012; Ward, 2011; Levy et al., 2016). Beyond the binary outcome, recruitment curves offer several additional advantages. By definition, recruitment curves assay MEPs at a range of increasing TMS intensities, and as such, illustrate a graded profile of CST function (Thickbroom et al., 2002). As a result, increases or decreases in slope or gain of the recruitment curve can signify recovery more closely than binary outcomes signaling the presence or absence of MEPs. For example, numerous studies have shown that decreases in recruitment curve parameters are indicative of more substantial CST damage, functional impairment, or poor recovery potential in patients with stroke (Devanne et al., 1997; Carroll et al., 2001; Liepert et al., 2005; Talelli et al., 2006; Ward et al., 2006; Lindberg et al., 2007; Lotze et al., 2012; Cunningham et al., 2014). In fact, graded increases in the slope of the recruitment curve have been associated with graded functional gains in recovery (Hummel et al., 2005) suggesting that metrics that are not binary, but rather based on an interval scale may serve as an effective monitor for rehabilitation-related recovery.

Recruitment curves are especially popular in stroke because they are believed to reflect CST gain and output from the region most linked to motor function, despite inherent damage, the primary motor cortex (M1; Devanne et al., 1997). However, given that other secondary motor cortices contribute to paretic hand function and recovery in stroke, it is possible that recruitment curves may also represent functioning of CST from higher motor areas beyond M1. For example, higher motor areas like the supplementary motor area (SMA) and premotor cortex (PMC) can support paretic hand function and recovery via re-mapping and plasticity changes proportional to the level of damage to CST from M1 (Weiller et al., 1992; Fries et al., 1993; Seitz et al., 1998; Liu and Rouiller, 1999; Fridman et al., 2004; Dancause et al., 2005; Ward et al., 2006, 2007; Bhatt et al., 2007; Takeuchi et al., 2007; Calautti et al., 2010; Zeiler et al., 2013; Plow et al., 2014). Indeed, SMA and PMC can offer alternate CST to the paretic upper limb, contributing in the range of 20–40% of entire CST (Dum and Strick, 1991; Schulz et al., 2012).

Understanding if there is a role of CST from secondary motor areas on recruitment curve properties is critical. TMS is already relevant for neurorehabilitation since it is simple and in-expensive. Therefore, by gaining this understanding, we could realize if using TMS generated recruitment curves could accurately and in-expensively interpret which areas re-map and contribute to overall CST function during recovery. For this reason, here we explored whether integrity of CST from PMC and SMA, besides M1, related to CST function as captured by recruitment curves in patients with chronic stroke. CST integrity was measured using diffusion tensor imaging (DTI) [fractional anisotropy (FA)] due to its long-standing use in neurology and generally accepted accuracy (Chenevert et al., 1990; Alexander et al., 2007; Soares et al., 2013). We argued that if recruitment curve properties were to reflect integrity of CST from higher motor cortices, then any increase in gain/output of the recruitment curve would signify their remapping in recovery. As such, our finding would create an opportunity to target PMC/SMA with techniques like cortical stimulation that are believed to boost recovery by boosting functioning of CST recovery (Fregni and Pascual-Leone, 2007). In addition, our results could help show that recruitment curves may serve as a simple, in-expensive means to assess function from areas generally studied with more resource-intensive structural and functional imaging in patients with stroke.

To our knowledge, only a pilot study by Lindberg et al. has directly investigated the relationship between CST integrity captured using DTI and recruitment curves in stroke. Within their study, Lindberg and colleagues found that a greater loss of integrity at the level of the cerebral peduncle was correlated with a reduced recruitment curve slope (Lindberg et al., 2007). However, because recent research has suggested that DTI indices describing CST integrity vary with extent and location of the lesion, it is critical to capture integrity not just in a single region but across several regions, and along the length of CST (Liepert et al., 2005; Zhu et al., 2010; Lindenberg et al., 2012; Schulz et al., 2012). Therefore, here, we chose to assess CST integrity at different regions along the path of CST. We captured FA at the most commonly used regions for analysis—the posterior limb of internal capsule (PLIC) and mean along the length of CST (Stinear et al., 2007; Allendorfer et al., 2012; Lindenberg et al., 2012). We also captured CST integrity in the region of the stroke lesion. We aimed to learn whether CST integrity at a specific region- PLIC, lesion or the length of CST pathways- closely related to neurophysiologic measurement of CST function described using the recruitment curve. We postulated that by identifying, which regions of CST most contribute to CST function, it would become possible to use recruitment curves as means to understand lesion characteristics, lesion load, and accordingly derive prognosis.

## Methods

### Participants

**Figure 1:**
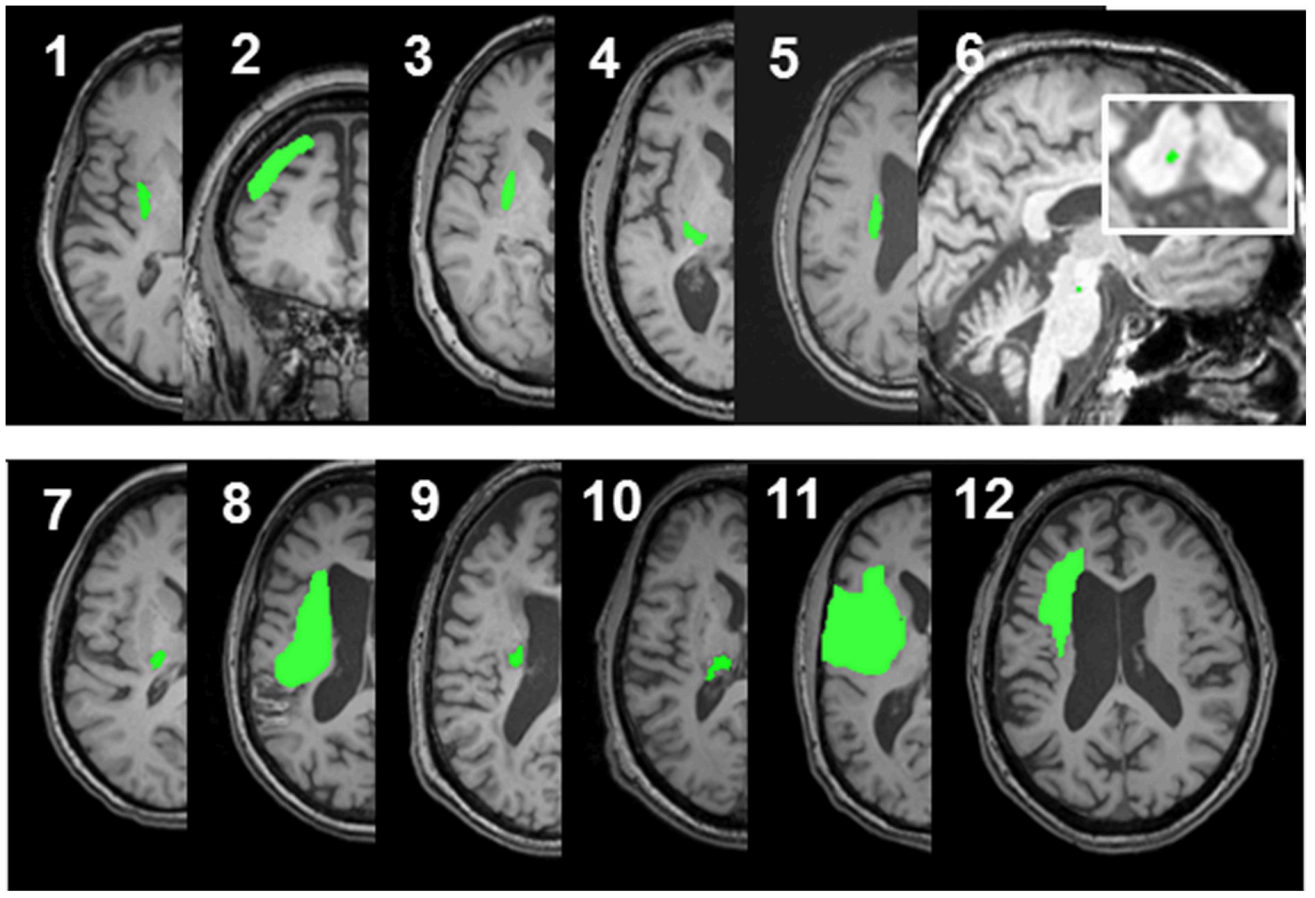
**Lesion locations for the 12 enrolled stroke patients**. Images were adjusted so the lesion would appear in the left hemisphere. Lesion for patient #6 is enlarged to demonstrate location and size. Lesion volumes are noted in Table 1.

Twelve patients who suffered a first-ever stroke, and eight healthy control subjects (68.3 ± 12.4 years) were enrolled (Table 1). Lesion locations for each patient are demonstrated in Figure 1. Patients were ≥21 years of age, in the chronic phase (>6 months) after a unilateral hemorrhagic or ischemic stroke and possessed at least a trace movement at the wrist, any of the fingers or the thumb of the paretic upper limb. Patients with contraindications to TMS, such as intracranial metallic implants, history of alcohol/substance abuse, seizures, neuro- and psycho-active medications lowering threshold for seizures, or cardiac pacemakers were excluded from the study, following published recommendations (Nitsche et al., 2008; Rossi et al., 2009; Shellock, 2014). All study procedures were approved by the Institutional Review Board (IRB) of the Cleveland Clinic Foundation. All participants provided written informed consent.

**Table 1:** **Patient Characteristics**.

**Patient**	**Age/Gender/Handedness**	**Edinburgh Handedness Inventory**	**Time Since Stroke (Months)**	**Side of Paresis**	**Stroke Location**	**Stroke Volume (cm^3^)**	**Stroke Subtype**	**Diabetic?**	**History of Smoking?**	**Medication Controlled Hypertension?**	**Gait Impaired?**	**Aphasia?**	**Stroke-Related PMC Damage**	**Stroke-Related M1 Damage**	**UEFM (Max 66)**	**% MSO at Ipsi MT (Max 100%)**
1	68/F/R	60	32	L	Basal Ganglia	2.2	Ischemic	N	N	N	Y	N	N	N	35	95
2	66/M/R	100	19	L	Cortical	9.4	Ischemic	N	N	Y	N	N	Y	Y	50	73
3	58/F/L	−40	24	L	Caudate Nucleus, Basal Ganglia	2.8	Ischemic	N	Y	Y	Y	N	N	N	54	76
4	69/M/R	100	23	R	Thalamus, Internal Capsule	1.5	Hemorrhagic	Y	Y	Y	Y	Y	Y	N	46	40
5	54/M/R	70	29	R	Caudate Nucleus, Basal Ganglia	2.4	Ischemic	N	N	Y	Y	Y	N	N	44	44
6	72/M/R	100	84	L	Pontine, Mesencephalus	0.01	Ischemic	Y	N	Y	Y	Y	N	N	59	40
7	55/F/R	80	48	R	Thalamus, Internal Capsule	1.8	Hemorrhagic	N	N	Y	Y	N	N	N	47	55
8	59/F/R	100	23	L	Temporal lobe, Basal Ganglia, Caudate Nucleus	80.8	Ischemic	N	N	N	N	Y	N	N	51	46
9	76/M/R	45.45	24	R	Basal Ganglia, Internal Capsule, Corona Radiata	0.9	Ischemic	N	Y	Y	Y	N	Y	N	15	100
10	50/M/R	100	54	L	Thalamus, Internal Capsule	1.0	Ischemic	N	N	Y	Y	Y	N	N	44	42
11	45/M/R	100	12	R	Temporal lobe, Internal Capsule	60.7	Hemorrhagic	N	Y	N	Y	Y	Y	Y	26	95
12	63/M/R	100	228	R	Striatum	48.6	Ischemic	N	Y	Y	Y	Y	Y	Y	53	48
Mean	61.3 8 (M) / 4 (F) 11 (R) / 1 (L)	76.3	50	6 (L) / 6 (R)		17.8	Ischemic (9) Hemorrhagic (3)	2 (Y)/ 10 (N)	7 (N) / 5 (Y)	3 (N) / 9 (Y)	10 (Y) / 2 (N)	7 (Y) / 5 (N)	5 (Y) / 7 (N)	3 (Y) / 9 (N)	43.3	67.3
St Dev	9.4	41.3	59.4			28.5									12.2	24.3

*%MSO at Ipsi MT denotes the stimulation intensity used to arrive at the RMT. Intensity is expressed as a % maximum stimulator output (MSO)*.

### Study design

**Figure 2:**
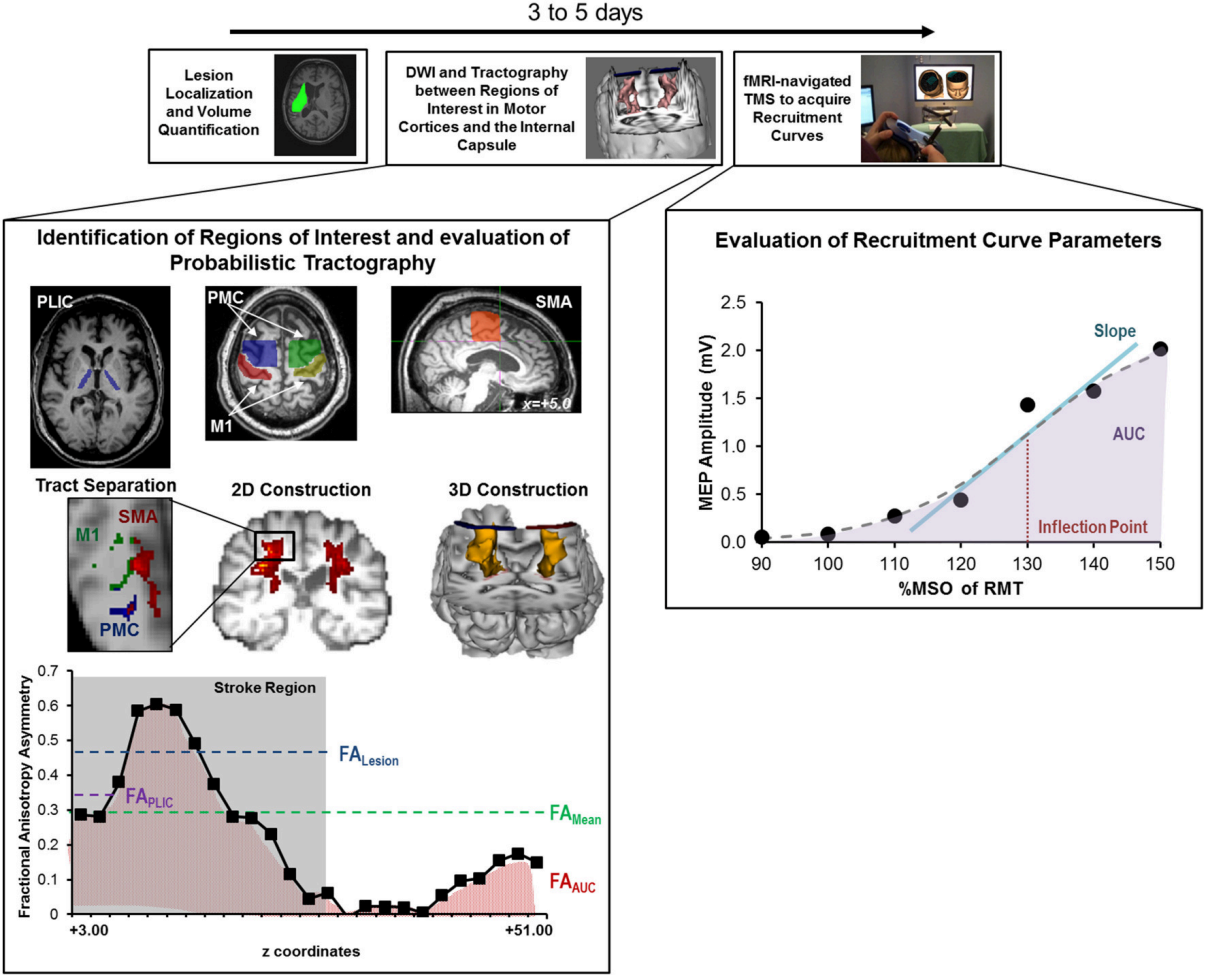
**Study design and quantitative analysis for assessing relationship between CST integrity assessed using DTI and recruitment curves captured using TMS. (Top)** General flow of experiments for the present study. **(Bottom Left)** Regions of interest were defined for the posterior limb of the internal capsule (PLIC), motor cortex (M1), premotor cortex (PMC), and supplementary motor area (SMA). Probabilistic tractography was performed between the PLIC and each respective motor cortex. Fractional anisotropy (FA) was determined along the length of the reconstructed CST and four indices of asymmetry between ipsilesional vs. contralesional CST were computed- FA_Lesion_, FA_PLIC_, FA_AUC_, and FA_Mean_. Indices are denoted in the figure at the bottom left, where FA_Lesion_ refers to the asymmetry index within the stroke region, FA_PLIC_ refers to the asymmetry index at the region of the PLIC, and FA_AUC_ and FA_Mean_ are a net or average asymmetry index for the determined CST. Coordinates denote the location of the brain/region of interest in respect to AC-PC alignment, where-in z = +0.00, denotes the level of alignment between the anterior commissure and posterior commissure. **(Bottom Right)** Recruitment curves acquired using TMS were modeled using the Boltzmann Equation and parameters of the curve (Slope, Inflection Point) were extracted for further comparisons. In addition, the area under the recruitment curve (AUC) was calculated using the trapezoidal area method. Recruitment curve slope and AUC were quantified as an asymmetry measure between the ipsilesional and contralesional hemisphere.

A schematic of the study design is shown in Figure 2 and study outcomes are diagrammed in Table 2. Clinical impairment was evaluated using the Upper Extremity Fugl Meyer score (UEFM), a commonly used scale that rates distal and proximal movements and upper limb coordination and reflexes on an ordinal scale (0–2) for a maximum score of 66 (Fugl-Meyer et al., 1975; Gladstone et al., 2002). Patients subsequently underwent magnetic resonance imaging (MRI) and TMS. T1-weighted MRI was used to quantify lesion locations and lesion volume. Diffusion-weighted/tensor imaging (DWI/DTI) was acquired to study CST integrity. In addition, patients underwent functional MRI (fMRI) during self-paced flexion-extension of the fingers of each hand. fMRI was acquired to provide for neuro-navigated TMS (Neggers et al., 2004) to help shorten testing and simplify thresholding needed to identify optimal site for TMS (details are provided in Cunningham et al., 2014). Specifically, given that substantial cortical remapping can occur, by employing fMRI-guidance, we aimed to add an additional layer of accuracy in our methodology (Lotze et al., 2006). Finally, fMRI-guided TMS was used to generate a stimulus-response or recruitment curve from the first dorsal interosseous (FDI) muscle.

**Table 2:** **Significance of Outcome Measures**.

**Analyzed metric**	**Measureable outcomes**	**Physiological representation**	**Significance of change in measurement**
Recruitment curve	Slope	Gain of descending CST	↓ Slope suggests a reduction in the overall strength of output of structurally available axonal tracts (Devanne et al., 1997)
	Area Under the Curve (AUC)	Overall CST output	↓ AUC suggests an overall reduction in CST recruitment and/or poor synchronization of descending volleys (Talelli et al., 2008)
	Fit to model (R^2^)	Accuracy of orderly CST recruitment	Fit values above 0.7 indicate orderly recruitment (*R* = 0.84; Carson et al., 2013)
	Inflection point	%RMT at which maximum gain is demonstrated	↓ Inflection point suggests loss of mid- and/or high threshold motoneuron populations and/or increased inhibitory components that alter recruitment (Talelli et al., 2006)
Fractional anisotropy (DTI)	FA_PLIC_	Average structural integrity of CST within the PLIC	↑ Fractional anisotropy suggests increased demyelination and axonal loss within the CST (at the respective measured location; Alexander et al., 2007)
	FA_Mean_	Average structural integrity of CST between the seed and target region of interest	
	FA_Lesion_	Average structural integrity of CST within the stroke region	
	FA_AUC_	Overall structural integrity of CST	

*FA, Fractional Anisotropy; CST, Corticospinal tract; AUC, Area Under the Curve; DTI, Diffusion Tensor Imaging*.

### Transcranial magnetic stimulation (TMS)

fMRI-guided, single pulse TMS (Magstim 200^2^, Wales, U.K.) was delivered using a figure-of-eight coil (diameter 70 mm; Cunningham et al., 2014). Individuals were seated comfortably in a chair that allowed them to rest their forearms and hands on a flat surface. MEPs were recorded in the FDI muscle using surface electromyography. EMG was acquired using bipolar Ag/AgCl electrodes (8 mm diameter) positioned over the muscle belly, with a reference electrode placed on the lateral epicondyle. All EMG signals were recorded using PowerLab 4/25 set at ± 10 mV, subsequently band-pass filtered (10–2000 Hz) and then recorded at a sampling rate of 4000 Hz.

Cranial landmarks (nasion, left ear, right ear) of each individual were registered with respective sites in the MRI via Brainsight. The voxel with peak fMRI activation in the region of M1 (or an adjacent site when M1 was damaged) was chosen as the initial site to study with TMS. Although the fMRI peak was used as an initial guide, we identified the optimal site for TMS using careful thresholding. The optimal site (motor hotspot) was determined by applying TMS across a 10 mm-resolution grid. The motor hot spot was defined as the site that evoked MEPs of at least 50 μV peak-to-peak amplitude in the FDI muscle in three out of five trials at the lowest TMS intensity. The intensity used to elicit criterion MEPs in the resting state of the muscle was called the resting motor threshold (RMT) commonly expressed as % maximum stimulator output, or %MSO (Rossini and Rossi, 2007). We confirmed that resting-state EMG activity was ≤ 10 μV in all patients and controls. Recruitment curves were acquired in resting state at the hotspot. Ten serial MEPs were collected at gradient increases in TMS intensity ranging from 90 to 150% of the RMT. Intensities were presented in a randomized order.

MEPs could not be generated from the ipsilesional hemisphere in the resting state of paretic FDI in patients 1, 9, and 11; the absence of resting-state MEPs in not uncommon in patients with severe loss of corticospinal output (Harris-Love et al., 2011). These patients were thus excluded from analysis of ipsilesional recruitment curves though they were included in the analysis of contralesional recruitment curves (Talelli et al., 2006).

Compound muscle action potentials or M-waves were acquired to normalize MEP data. Maximum M-waves (M-MWaves) were elicited by applying a supramaximal electrical stimulus to the ulnar nerve at the wrist. Electrodes (Ultratrace 1690) were placed at the distal end of the ulnar nerve ~2 inches away from the wrist. Increasing stimulus intensities were applied ranging from 1 to 15 mV for 0.5 to 1.5 ms until a maximum EMG response was noted.

### Diffusion tensor imaging (DTI)

DTI was used to quantify CST integrity. Here, a High Angular Resolution Diffusion Weighted Imaging (HARDI-DWI) dataset was acquired on a Siemens 3T TIM Trio with 71 diffusion-weighting gradients (*b* = 1000 s/mm^2^) and 8 image volumes (*b* = 0 s/mm^2^) for a total scan time of 12 min. Each DWI scan allotted for whole brain coverage and 2-mm isotropic voxels (field of view: 256 × 256 mm, image matrix: 128 × 128, and 52 2-mm thick slices).

### Data analysis

#### Lesion volume and location

The anatomical location of each patient's stroke lesion was determined on T1-weighted MRI images by a trained neurologist (AM). Lesion volume was defined using MRIcro, a free, readily-downloadable software (http://www.mccauslandcenter.sc.edu/mricro/). Lesion volumes were found using manually drawn regions of interest (ROI) along a single plane of the stroke lesion (Zhu et al., 2010).

#### TMS analysis

Recruitment curves were plotted as intensity (90–150% RMT) vs. MEP size noted as peak-to-peak amplitude (expressed as raw values in mV and %M-MWave; Figure 2) (Rossini and Rossi, 2007). For each intensity, MEP size was averaged across all 10 TMS trials. Two main parameters were computed: area under the recruitment curve (RC_AUC_) and slope (RC_Slope_). For analysis of the ipsilesional hemisphere, nine patients were studied whereas, 12 patients were evaluated for the contralesional hemisphere.

##### *RC_*AUC*_: overall CST output*.

RC_AUC_ values for each hemisphere were quantified using the trapezoidal area method (Figure 2). RC_AUC_ for the ipsilesional hemisphere was expressed relative to RC_AUC_ of the contralesional hemisphere following Equation 1, with values <1 indicating a RC_AUC_ for the ipsilesional hemisphere. We chose to normalize values to the contralesional hemisphere to control for inter-subject differences (Lindberg et al., 2007; Lotze et al., 2012).

(1) $$\begin{array}{l} RC\;AUC_{\;Ipsi/Contra\;}=\;\frac{Ipsilesional\;RC\;AUC\;}{Contralesional\;RC\;AUC\;}\; \end{array}$$

##### *RC_*Slope*_: descending CST gain*.

To quantify the gain of the stimulus-response curve, all recruitment curves were fitted, as described previously (Devanne et al., 1997; Kaelin-Lang and Cohen, 2000; Carroll et al., 2001; Carson et al., 2013), using a nonlinear sigmoidal model (Boltzmann Equation) shown in Equation 2.

(2) $$\begin{array}{l} y=\;\frac{A_{t}\;-\;A_{b}}{1+\;e^{\left(-\frac{x-x_{0}}{w}\right)}}\;\; \end{array}$$

The function parameters *A*_*t*_ and *A*_*b*_ denote the asymptotic *y*-values of the sigmoidal function, where the x-range of the sigmoidal slope was defined as *w* and the midpoint of the slope as *x*_*o*_. The *x*-values were taken to be the %RMT, ranging from 90 to 150%. Therefore, the four function parameters were adjusted to best fit the modeled *y*-value to the experimental *y*-value. The slope of the sigmoidal curve, the midpoint (inflection point) and *R*^2^ of the fit were recorded (Figure 2; Table 2). A fit above 0.7 was considered to be indicative of an accurate model (Carson et al., 2013). The inflection point was defined as the point of 50% of the maximal MEP for each patient (Table 2). Similar to the AUC, RC_Slope_ of the ipsilesional hemisphere was expressed relative to RC_Slope_ of the contralesional hemisphere following Equation 3.

(3) $$\begin{array}{l} RC\;Slope_{Ipsi/Contra}=\frac{Ipsilesional\;Slope}{Contralesional\;Slope} \end{array}$$

#### DTI analysis

DTI images were corrected for eddy currents and head motion using FSL (Jenkinson et al., 2012). Whole brain diffusion tensor maps of FA were calculated by first least-squares fitting of the 71 acquired diffusion profiles to each of the six independent tensor elements and then determining the final tensor-based value. Fiber orientation distribution functions (FOD) were used to account for crossing fibers on the 71 acquired diffusion profiles, as previously described by our group (Sakaie and Lowe, 2007; Lowe et al., 2008). Prior to tractography, we applied a threshold value of 0.2 on all FA maps. A threshold value of 0.2 has been used extensively. It is believed to be an optimal level to ensure that all ROI in the brain, including the centrum semiovale (known to have inherently low FA values) remain in the FA skeleton in patients with stroke (Kunimatsu et al., 2004; Zhu et al., 2010).

CSTs were virtually reconstructed using a three-dimensional random walk probabilistic tracking method (Sakaie and Lowe, 2007; Lowe et al., 2008; Zhang et al., 2013). Briefly, the track density value of each voxel was used as the probability distribution to generate stepping directions. Structural masks of the right and left hemisphere were applied during tracking and tracts branching outside the brain tissue hemisphere mask were terminated and not included in analysis. Our probabilistic tractography was constrained to voxels with more than 95% of the individual tract-specific connectivity probability, wherein voxels outside of the 95th percentile were assumed to have a track density value of zero. A “slice-by-slice” analysis was established by calculating a mean FA across all non-zero voxels at each z-slice (Lowe et al., 2008).

Thresholded probabilistic tracking was performed from the PLIC to the M1, PMC or SMA separately. Seeds were defined at the level of the PLIC, since the internal capsule is mainly comprised of CST which control voluntary movement as opposed to other tracts such as the corticobulbar (Holodny et al., 2005). ROI for PLIC were defined directly on the axial plane of the FA map at the appropriate level of the foramen of Monroe. ROIs for M1, PMC, and SMA (Figure 2) were drawn on ACPC aligned T1-weighted images based on guidelines (Bhatt et al., 2007) and then transformed into DTI (*b0*) space. Errors due to between-space transformations were corrected manually.

Structural integrity of CST originating from the M1, SMA, or PMC was compared between the ipsilesional and contralesional hemispheres using the asymmetry index of FA (described in Equation 4). The asymmetry index is given by Equation (4), where values range from −1 to +1; values >0 denoted increased CST damage on the ipsilesional side. For control subjects, the right hemisphere (non-dominant) was considered the ipsilesional side and the left hemisphere (dominant) was the contralesional.

(4) $$\begin{array}{l} FA_{asymmetry}=\;\frac{FA_{contralesional}\;-\;FA_{ipsilesional}}{FA_{contralesional}\;+\;FA_{ipsilesional}} \end{array}$$

Since CST integrity can be influenced by stroke location, overlap with the lesion, and degeneration, we determined FA asymmetry values for CST integrity at several different regions (Figure 2). First, we computed the average FA asymmetry along the entire length of the tract (termed FA_Mean_). Second, a FA index was determined at the region of the PLIC (termed FA_PLIC_), which represents one of the most common forms of analysis (Stinear et al., 2007). Here, PLIC was defined at the level of three consecutive axial slices along the CST (Sidaros et al., 2008; Puig et al., 2011; Park et al., 2013). Third, the entire area under the FA asymmetry curve was calculated using the trapezoidal rule (FA_AUC_) in Matlab (Mathworks, Inc.). Finally, we evaluated FA asymmetry within the stroke region (termed FA_Lesion_; Grandin et al., 2001; Granziera et al., 2007, 2010) because substantial reductions in CST integrity occur near the lesion site (Lindenberg et al., 2012). To help determine FA_Lesion_ an expert neurologist (AM) localized the region of stroke on z-slice levels for each patient. A second blinded rater (EP) repeated the analysis with excellent inter-rater reliability [ICC(2) = 0.925]. The mean FA asymmetry across the stroke region and mirror region in the contralesional hemisphere was recorded. For all DTI comparisons, patient 6 was excluded since their stroke (pontine/mesencephalic) was outside of the investigated CST region and could potentially affect the accuracy of FA measures within the PLIC due to retrograde degeneration (Kobayashi et al., 2005; Liang et al., 2008; Schulz et al., 2012).

### Statistical analysis

Statistical analysis was performed using Statistical Package for the Social Sciences (SPSS Inc.). All data was tested for normality using the Shapiro–Wilk test. For normally distributed data, a student's *t*-test was utilized to analyze differences between patients and controls. For non-normal data, the Mann–Whitney *U*-test was used to determine differences between patients and controls. Corrections for multiple comparisons were incorporated when applicable. For all comparisons between healthy and stroke patients, comparisons were only examined between the ipsilesional and right control hemisphere (non-dominant), and the contralesional and left control hemisphere (dominant; Cunningham et al., 2015).

We utilized a repeated-measures analysis of variance (ANOVA) with polynomial contrast to determine differences between FA asymmetry collected at different regions (FA_PLIC_, FA_Mean_, FA_AUC,_ and FA_Lesion_) in patients with stroke. If Mauchly's test of sphericity was violated, we applied a Greenhouse-Geisser correction for final *F*-Values. To identify if FA asymmetry at which particular regions was different, we used pair-wise comparisons with a Bonferroni confidence interval adjustment.

We examined the bivariate correlation between FA asymmetry values and parameters of the recruitment curves (RC AUC_Ipsi/Contra_, RC Slope_Ipsi/Contra_) using the Pearson's correlation coefficient. Based on the Pearson's correlation coefficient criteria, a small (0.1–0.3), medium (0.3–0.5), or large (0.5–1) association was determined. Inter-rater reliability was determined using intraclass correlation coefficients (ICCs). ICCs were calculated using a two-way random-model with consistency agreement. ICC values >0.8 were defined as excellent agreement between raters (Danielian et al., 2010). All utilized tests were two-sided, where *p* ≤ *0.05* was considered statistically significant.

## Results

### Clinical assessment

Patient characteristics are presented in Table 1. Age differences between controls (68.3 ± 12.4 years) and patients (61.3 ± 9.4 years) were not significant (Table 1; *t* = 1.44, *p* = 0.166). All controls and 11 of the 12 patients were right handed as determined by the Edinburgh Handedness Inventory (EHI; EHI Patients = 76.3 ± 41.3; Oldfield, 1971). The average lesion volume was 17.67 ± 8.22 cm^3^ (s.e.m.), where strokes affecting the basal ganglia (*n* = 5) or PLIC (*n* = 5) were most common. The average UEFM score was 43.3 ± 12.2 (range 15–59).

### Recruitment curve properties in patients with chronic stroke

Average RMT for the ipsilesional hemisphere (67.3 ± 24.3%) was significantly higher than RMT for the contralesional hemisphere (46.6 ± 11.4%; *t* = 2.667; df = 18; *p* = 0.014) and RMT for controls (42.8 ± 6.1%; *t* = −2.773; df = 18; *p* = 0.013). M-Mwaves were also not different between patients and controls [ipsilesional (*t* = 1.254; df = 18; *p* = 0.226) and contralesional (*F* = 3.405; *t* = −1.152; df = 18; *p* = 0.265)].

**Figure 3:**
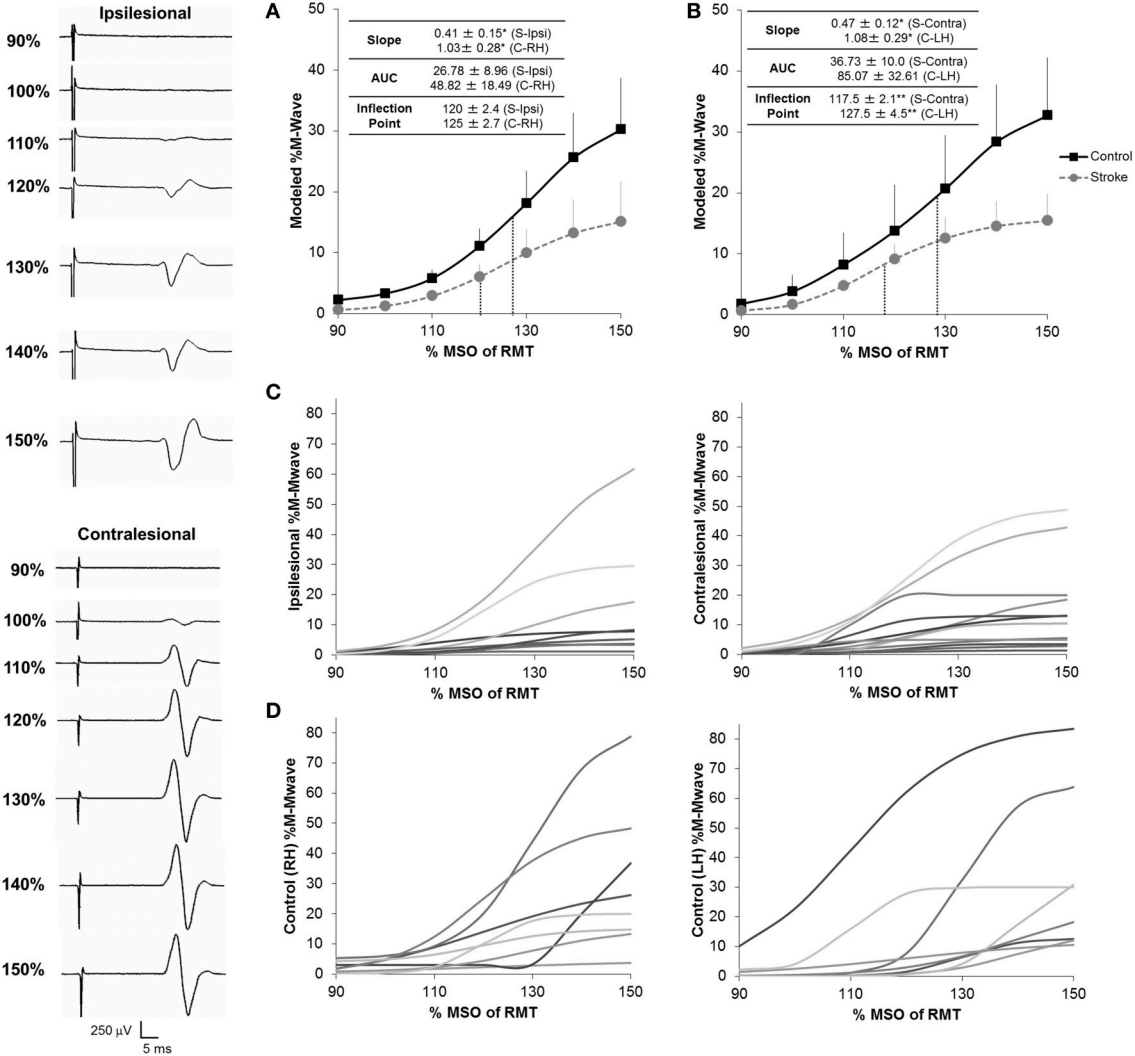
**Recruitment curves for stroke subjects and healthy controls**. We noted a significant reduction in the gain of descending CST, as shown by the slope of the recruitment curve, between stroke and healthy controls in both the ipsilesional (*n* = 9) **(A)** and contralesional (*n* = 12) **(B)** hemispheres. In addition, the contralesional hemisphere displayed a reduced inflection point in comparison to controls suggesting either loss of higher threshold motoneuron populations and/or increased inhibitory components in this hemisphere. No significant differences for overall CST output, as shown by the area under the curve (AUC) were noted between stroke patients and controls in either hemisphere. Inflection points are shown in blue dashes for all conditions. Data in the ipsilesional hemisphere is only shown for patients eliciting a resting state recruitment curve (*n* = 9), since muscle activation can influence recruitment curve gradients. In contrast, since all patients elicited a resting state recruitment curve in the contralesional hemisphere, all patients are presented in panels **(B,C)**. Data was averaged across each subject population following mathematical modeling, normalized to the max %Maximum MWave (M-Mwave) and plotted ± s.e.m. for each assessed intensity. Recruitment curves are presented for all patients with stroke **(C)** and controls **(D)**. Representational motor evoked potentials from patient 10 are shown to the left of the plotted data. ^*^p = 0.05; ^**^p = 0.07 for control vs. stroke. S, Stroke; Ipsi, Ipsilesional; Contra, Contralesional; RH, Right Hemisphere; LH, Left Hemisphere.

With regards to recruitment curves, controls had an average fit (*R*^2^) of 0.77 ± 0.072 and 0.95 ± 0.007 in the right and left hemispheres and patients had a fit of 0.80 ± 0.06 and 0.82 ± 0.05 in the ipsilesional (*n* = 9) and contralesional (*n* = 12) hemispheres. Interestingly, fit accuracy was significantly different between hemispheres in healthy controls (*U* = 4.5; *Z* = −2.89; *p* = 0.004) due partially to hand dominance (Wittenberg and Schaechter, 2009). Recruitment curve parameters in patients significantly differed from those in healthy controls (Figure 3). We noted a significantly reduced RC_*Slope*_ in the ipsilesional (*U* = 16; *Z* = −1.925; *p* = 0.05) and contralesional (*U* = 21; *Z* = −2.083; *p* = 0.03) hemispheres (Figures 3A,B; Table 2). No significant differences in the inflection point were noted between controls and patients. In addition, while we noted a slight reduction in RC AUC between patients and controls, significance was not reached between groups. Taken collectively, we noted that patients demonstrated reduced CST gain (RC_Slope_) and output (RC_AUC_) in comparison to controls. Recruitment curves for all stroke subjects and controls are shown in Figures 3C,D, respectively.

### CST integrity

**Figure 4:**
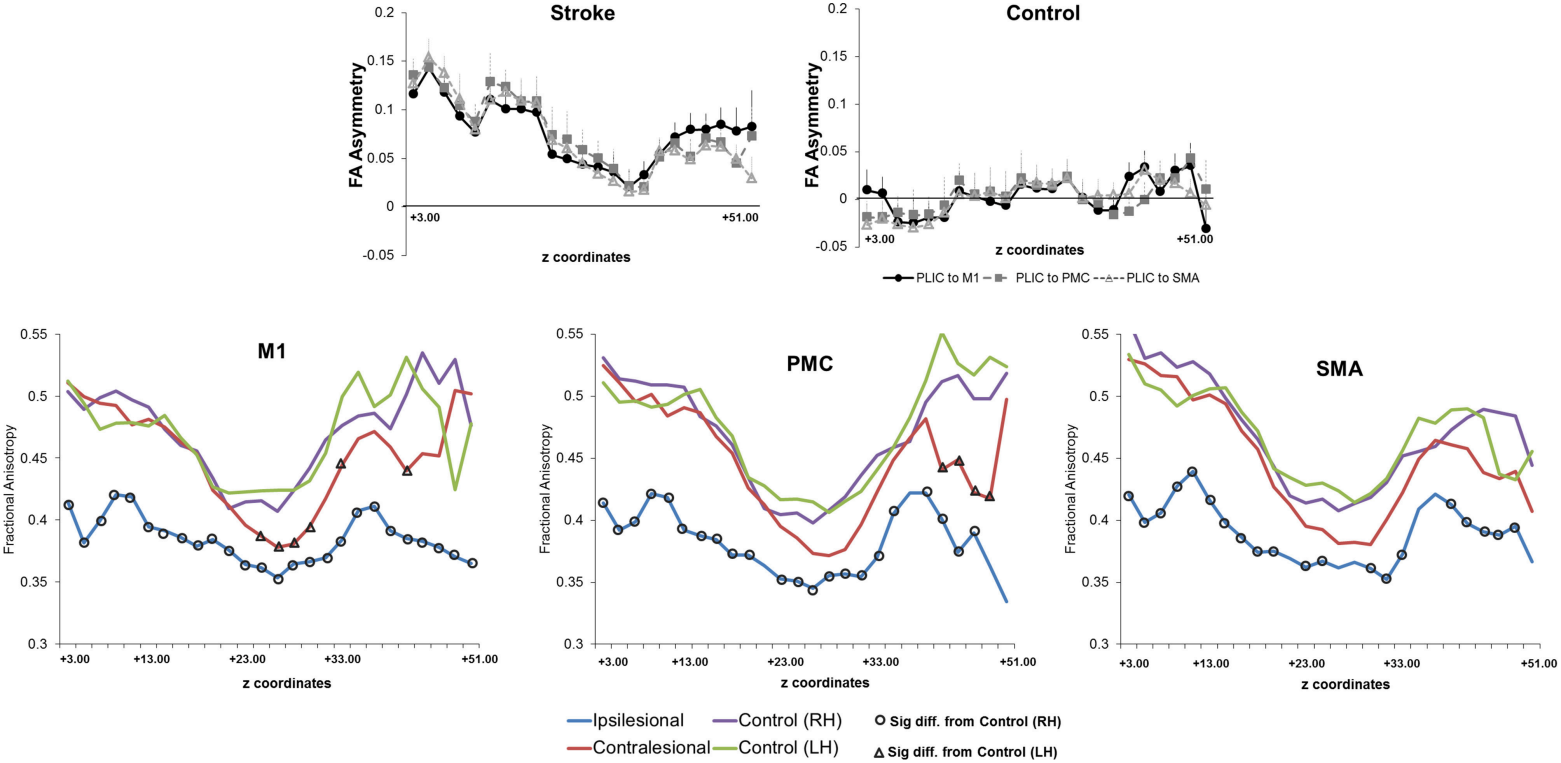
**Fractional anisotropy and fractional anisotropy asymmetry across three sets of corticospinal tracts (CSTs) in patients with stroke and healthy controls. (Top)** Average CST tractography FA asymmetry values between seed and target(s) (*n* = 11). Higher values of FA asymmetry, indicative of greater CST damage, were noted along the entire tract in comparison to healthy controls. The CST integrity between tracts descending from the M1, PMC, or SMA was similar in either patients or controls (*n* = 8). **(Bottom)** Raw fractional anisotropy values were plotted along the length from the M1 **(left)**, PMC **(middle)**, and SMA **(right)**. Patients displayed significantly higher levels of CST damage, as indicated by a decreased area of fractional anisotropy, in both the ipsilesional (differences are shown with black circles) and contralesional (differences are shown with black triangles) hemispheres in comparison to controls. Significance was defined as *p* = 0.05. Coordinates denote the location of the brain/region of interest in respect to AC-PC alignment, where-in z = +0.00, denotes plane of the anterior commissure and the posterior commissure.

**Figure 5:**
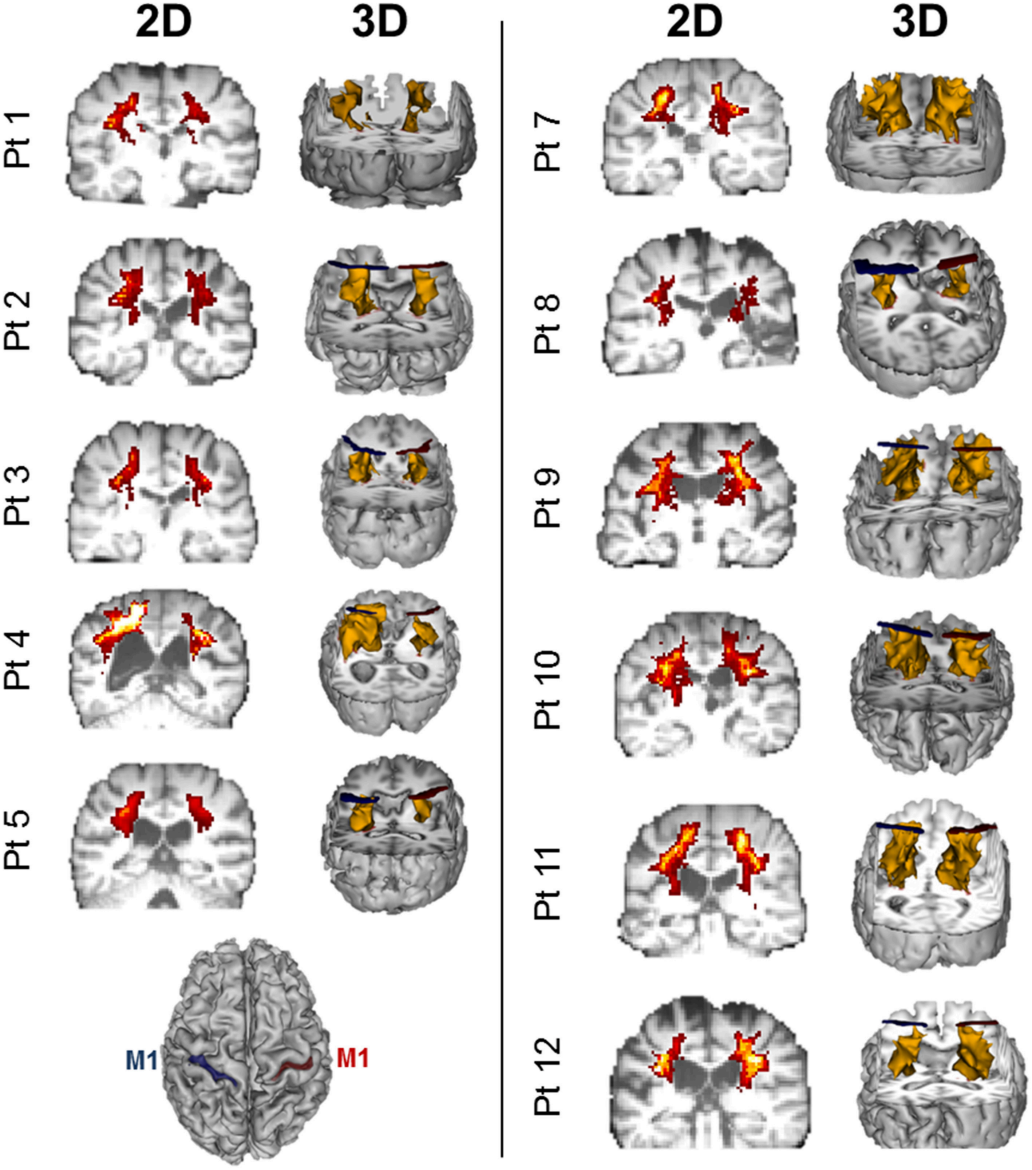
**Diffusion weighted imaging (DWI)-based probablistic tractography across patients from primary motor cortex to posterior limb of the interal capsule**. Probablistic tractography was performed across all patients from the posterior limb of the internal capsule (PLIC) to the primary motor cortex (M1) for each hemisphere. Regions of interest for tractography were defined as outlined in Figure 2 and Section Statistical Analysis. Two dimensional (2D) corticospinal tract (CST) density maps are shown for each patient, with yellow/orange denoting more dense CSTs. 3D reconstruction of tractography is also displayed for each patient in yellow. Blue ROIs denote the left hemisphere M1 (see **bottom left**), while red ROIs denote the right hemisphere M1.

Next, we assessed CST integrity for tracts descending from the M1, PMC, and SMA. For these sets of tracts, we studied FA asymmetry in different regions (FA_PLIC_, FA_Mean_, FA_AUC_, FA_Lesion_), and raw FA diffusivity values (Figure 4). Visual reconstructions of tractrography are demonstrated in Figures 2, 5.

We first compared CST integrity (FA asymmetry) across tracts descending from the M1, PMC, and SMA. Though differences were not significant in controls, in patients, integrity in the region of the lesion differed [FA_Lesion_ (*F*_(1.833, 20.159)_ = 6.942; *p* < 0.006)]. CST from M1 demonstrated significantly more damage at the region of the lesion (FA_Lesion_) in comparison to CST from PMC (*p* = 0.04) and SMA (*p* = 0.02); albeit noted differences were minor, with an average difference in means of 0.015. Next, we compared CST integrity (FA asymmetry) between patients and controls. We found increased FA asymmetry in patients across all regions of analysis- FA_PLIC_, FA_Mean_ and FA_AUC_, and FA_Lesion_. FA asymmetry differed based on which region was studied in patients. We found significant differences between FA_Lesion_ and FA_Mean_, and FA_PLIC_ and FA_Mean_ for CST descending from the M1 [*F*_(2, 22)_ = 9.826; *p* < 0.001], PMC [*F*_(2, 22)_ = 19.660; *p* < 0.0001], and SMA [*F*_(2, 22)_ = 14.838; *p* < 0.0001; all *p* < 0.05; Table 3]. Therefore, CST integrity denoted as FA_Lesion_ and FA_PLIC_ was indicative of most damage. Besides FA asymmetry, we compared values of raw FA between patients and controls (see Table 4 for values). We found that raw FA was reduced along the majority of CST from ipsilesional M1, PMC and SMA in patients vs. controls (all *p* < 0.05; Figure 4). In addition, we found that the contralesional hemisphere showed regions of reduced FA in CST descending from M1 and the PMC in comparison to controls (all *p* < 0.05; Figure 4; Buffon et al., 2005; Schaechter et al., 2009; Crofts et al., 2011; Dacosta-Aguayo et al., 2014).

**Table 3:** **Fractional Anisotropy (FA) asymmetry indices in patients with stroke (*n* = 11) and healthy controls (*n* = 8)**.

**Patient group**				**Control group**			
**Parameter**	**M1**	**PMC**	**SMA**	**Parameter**	**M1**	**PMC**	**SMA**
FA_PLIC_	0.126 ± 0.015^a^	0.134 ± 0.014^a^	0.140 ± 0.015^a^	FA_PLIC_	0.009 ± 0.019	−0.018 ± 0.018	−0.027 ± 0.018
FA_Mean_	0.078 ± 0.009^b^	0.076 ± 0.012^b^	0.065 ± 0.01^b^	FA_Mean_	0.005 ± 0.003	0.004 ± 0.004	−0.002 ± 0.003
FA_Lesion_	0.096 ± 0.01	0.112 ± 0.012	0.109 ± 0.009	FA_Lesion_	—	—	—
FA_AUC_	1.773 ± 0.255	1.791 ± 0.323	1.653 ± 0.234	FA_AUC_	0.074 ± 0.055	0.112 ± 0.092	−0.018 ± 0.079

a *Significantly different from FA_Mean_ (p ≤ 0.01)*.

b *Significantly different from FA_Lesion_ (p ≤ 0.04)*.

**Table 4:** **Non-normalized (raw) fractional anisotropy (FA) diffusivity values for patients with stroke and healthy controls**.

**Ipsilesional (Patient)**				**Contralesional (Patient)**			
**Parameter**	**M1**	**PMC**	**SMA**	**Parameter**	**M1**	**PMC**	**SMA**
FA_PLIC_	0.394 ± 0.014	0.394 ± 0.013	0.397 ± 0.014	FA_PLIC_	0.505 ± 0.009	0.514 ± 0.013	0.526 ± 0.015
FA_Mean_	0.382 ± 0.008	0.379 ± 0.008	0.386 ± 0.007	FA_Mean_	0.448 ± 0.008	0.445 ± 0.009	0.443 ± 0.010
FA_Lesion_	0.387 ± 0.009	0.379 ± 0.011	0.387 ± 0.011	FA_Lesion_	—	—	—
**Right Hemisphere (Control)**				**Left Hemisphere (Control)**			
FA_PLIC_	0.487 ± 0.018	0.508 ± 0.019	0.537 ± 0.018	FA_PLIC_	0.491 ± 0.014	0.498 ± 0.016	0.517 ± 0.018
FA_Mean_	0.459 ± 0.012	0.464 ± 0.013	0.468 ± 0.014	FA_Mean_	0.465 ± 0.009	0.468 ± 0.012	0.468 ± 0.013

### Relationship between recruitment curves and CST integrity

**Figure 6:**
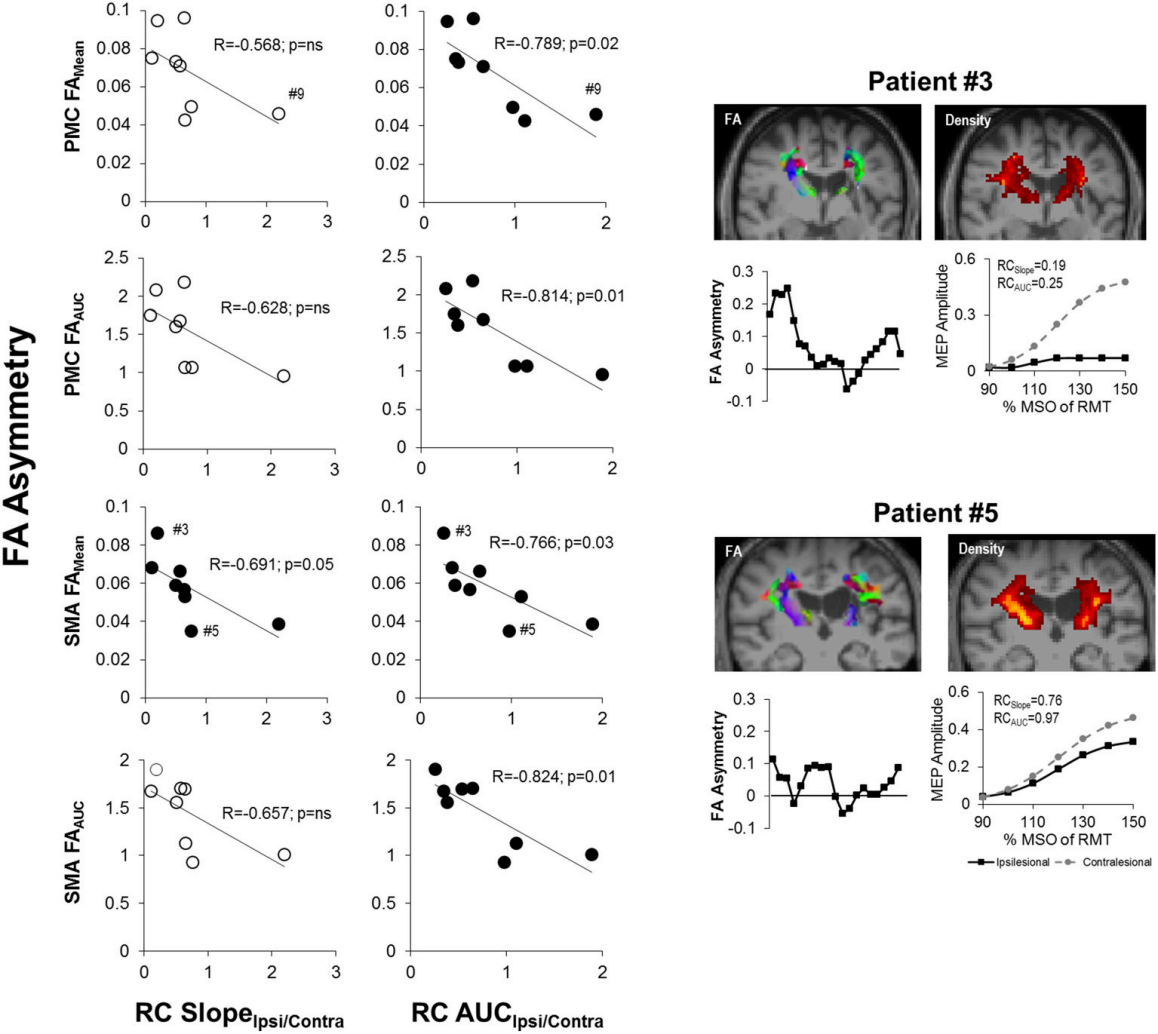
**Recruitment curve slope and area under the curve predicts level of corticospinal tract (CST) damage in patients with stroke. (Left)** The gain of descending CST, as indicated by the slope (RC Slope_Ipsi/Contra_), was found to be significantly negatively correlated with the average structural integrity of CST (FA_Mean_) for CST descending from the SMA (*p* = 0.05; *n* = 8). **(Middle)** The area under the recruitment curve, a representation of the overall output of stimulated CST (RC AUC_Ipsi/Contra_), however, had an even stronger relationship with CST damage. Specifically, RC AUC_Ipsi/Contra_ was significantly negatively correlated with overall CST integrity (FA_Mean_ and FA_AUC_) for CST originating from either the PMC or SMA (*p* = 0.03). Removal of Patient #9 from analysis did not change observed results. Filled circles denote significant relationships. **(Right)** Sample data of stroke patient with severe CST damage (upper; Patient #3) in comparison to patient with moderate damage (lower; Patient #5), as marked by #3 and #5 in SMA FA_Mean_ plots, and their respective FA asymmetry and recruitment curves. Fractional anisotropy maps (FA) are shown for each patient. Red denotes fibers in the x-axis (left to right), green denotes fibers in the y-axis (anterior to posterior) and blue denotes fibers in the z-axis (superior to inferior). CST density maps are shown in the top right for each patient, with yellow/orange denoting more dense CSTs.

We next examined the relationship between recruitment curves and integrity of CST from M1, PMC and SMA in patients. Notably, we found that RC AUC_Ipsi/Contra_ was related with FA asymmetry (*p* = 0.05) of tracts from PMC and SMA but not of M1. Specifically, as depicted in Figure 6, RC AUC_Ipsi/Contra_ (*n* = 8) was negatively correlated with FA_Mean_ and FA_AUC_ for CST from the PMC and the SMA. RC Slope_Ipsi/Contra_ too was only related to FA asymmetry for tracts from SMA but not from M1. In particular, we found only one significant correlation between RC Slope _Ipsi/Contra_ and FA_Mean_ for CST descending from the SMA. Taken collectively, we observed that patients who had both a reduced gain (RC Slope_Ipsi/Contra_) and reduced output from the recruitment curve (RC AUC_Ipsi/Contra_) presented with high levels of CST damage within the PMC and SMA, as illustrated in Figure 6.

**Figure 7:**
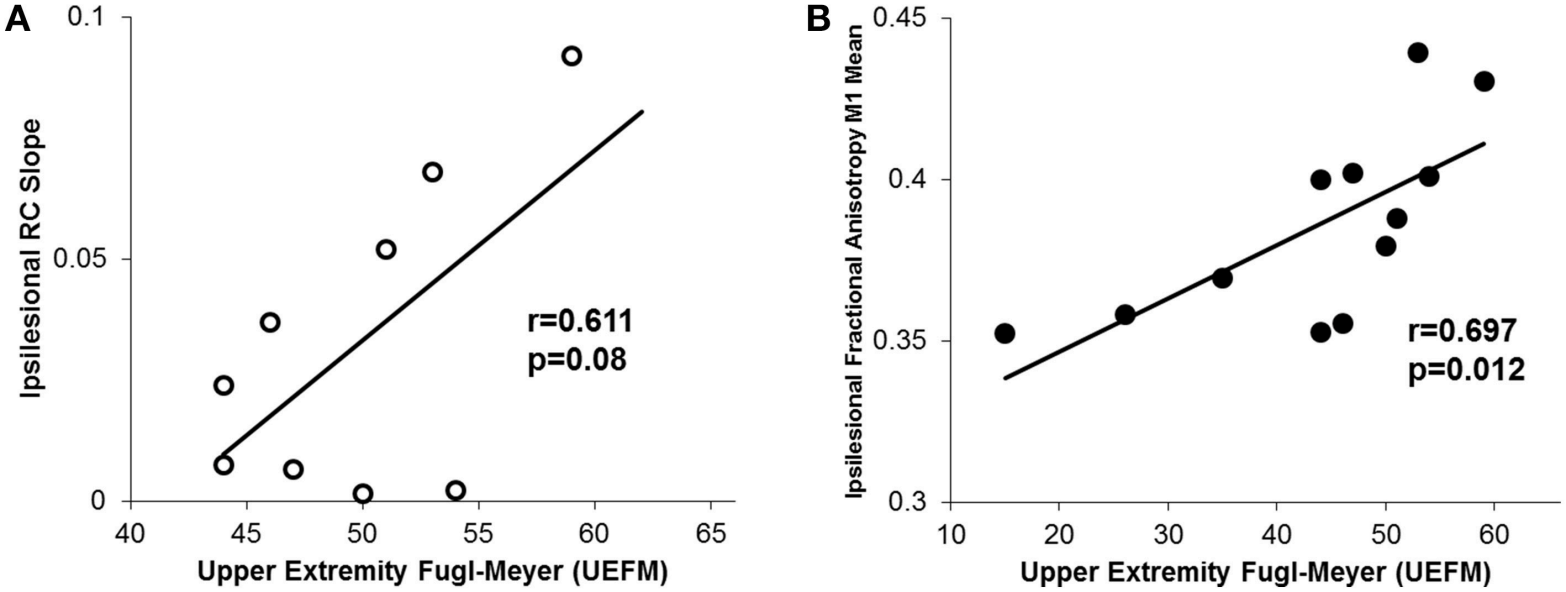
**Relationship between motor impairment (UEFM) and CST integrity and recruitment curves. (A)** We observed a trending positive relationship between the ipsilesional recruitment curve slope and upper extremity fugl-meyer (UEFM). **(B)** Similarly, a significant positive correlation was noted between UEFM and the ipsilesional mean raw fractional anisotropy in CST from M1. Higher fractional anisotropy values here denote a more intact tract.

Finally, similar to other reports, we noted that higher UEFM (less impairment) was significantly correlated with less CST damage of ipsilesional tracts and a trending increase in ipsilesional recruitment curve slope (Figure 7; Ward et al., 2006; Lindberg et al., 2007; Stinear et al., 2007, 2012).

## Discussion

The goal of the present study was to assess if recruitment curves reflect integrity of CST from motor regions beyond M1 in patients with chronic stroke. Specifically, we aimed to determine whether CST descending from the PMC and SMA, in addition to M1, made unique contributions to recruitment curve properties. The main findings from our study show that output of the recruitment curve (RC AUC_Ipsi/Contra_) is most intimately associated with CST integrity from the premotor areas but not the primary motor cortex (M1). Association varied based on which regions of CST integrity were investigated (Table 2). For example, recruitment curve output was most related to CST integrity measured along the entire length of CST (FA_AUC_ and FA_Mean_), but not integrity studied at specific regions, like internal capsule or lesion territory (FA_Lesion_ and FA_PLIC_). Based on our observations, recruitment curves could sensitively capture re-mapping of function to higher motor cortices, and help comprehensively infer damage and degeneration that occurs typically along the length of CST in chronic recovery. As such, future studies should explore whether TMS-based recruitment curves can serve as a less expensive, and easy-to-administer proxy for functional and structural imaging in stroke.

### Contribution of higher motor areas to recruitment curve properties

We have identified that in patients with chronic stroke, CST function captured by recruitment curves (RC AUC_Ipsi/Contra_) is most representative of integrity of CST from higher motor areas (PMC and SMA). This finding is conceivable given that higher motor areas re-map to contribute to recovery in chronic stroke (Seitz et al., 1998; Fridman et al., 2004; Dancause et al., 2005; Ward et al., 2006; Plow et al., 2014). Indeed, re-mapping has been shown to increase with damage to territories in M1 and loss of its CST (Weiller et al., 1992; Seitz et al., 1998; Bhatt et al., 2007; Ward et al., 2007). Thus, in our sample, since raw FA diffusivity of CST indicated greater damage to CST from M1 than that to CST from PMC and SMA (Figure 5), it is explainable that recruitment curve properties were related to residual CST from PMC and SMA but not damaged CST from M1. Our results suggest that chronic stroke patients may rely on intact CST from re-mapped territories in higher motor areas to elicit output in paretic muscles.

Our results are also possible given that TMS can excite higher motor areas via cortico-cortical projections from M1 (Lemon, 1999; Klöppel et al., 2008). Indeed, both animal experiments and human studies have suggested that cortico-cortical projections between the premotor-motor cortices remain an important mechanism for trans-synaptic excitation of fast-conducting pyramidal cells in M1 (Ghosh and Porter, 1988; Klöppel et al., 2008). However, recruitment of higher motor areas, whether through remapping or through cortico-cortical connections, would not result in complete motor recovery given that they project CST that contain polysynaptic, less-myelinated, slow-conducting axons than M1 (Boudrias et al., 2006; Ward, 2011). For example, our patients had a UEFM of 43.3 ± 12.2 (max = 66) indicating that they were moderately affected, but were still in chronic recovery (Duncan et al., 1983). Thus, role of higher motor areas could be a reasonable proxy given that CST from M1 are damaged most commonly (Bogousslavsky and Regli, 1990; Johansen-Berg et al., 2002; Buffon et al., 2005).

An interesting finding was that the gain of the recruitment curve (RC Slope_Ipsi/Contra_) was only associated with integrity of CST descending from the SMA. One possibility for this result is the hierarchy of CST recruitment. Traditionally, motor unit recruitment following TMS begins with low-threshold, large diameter CST emerging from M1 (Devanne et al., 1997; Ward et al., 2006). Then, as the stimulus intensity increases, higher-threshold, small diameter fibers, such as those from the PMC and then SMA are recruited (Devanne et al., 1997; Henderson et al., 2006). Given this order, damage of select high-threshold fibers in the SMA, which may occur with more extreme damage (Lukács et al., 2008), could eventually influence the slope of the recruitment curve and consequently, motor function.

#### Other factors explaining contribution of higher motor areas to recruitment curve properties

Some could argue that our result that recruitment curves represent CST function from anteriorly located higher motor territories is surprising given that the location of the motor hotspot, most likely in M1, did not significantly differ between patients and controls (data not shown). However, we note that beyond neurophysiologic influence, several other factors may have affected our observed results. First, TMS using a figure-eight coil is inherently non-focal. Indeed, it has previously been shown that while the electrical field strength is maximal under the middle of the coil, the spatial derivative of the electric field is also highest below the center of each lobe (Civardi et al., 2001). Thus, fibers aligned between the middle of the coil and the center of the anteriorly directed lobe may have become preferentially activated (MacCabee et al., 1993). Therefore, while M1 was likely activated at the hotspot by TMS, inherent diffuse electric field spreads that would occur within ~15 mm from the middle of the coil may have activated other structures like the PMC and SMA (MacCabee et al., 1993). Second, several of our patients (*n* = 5) had characteristic damage to the posterior part of their PLIC (Kobayashi et al., 2005; Liang et al., 2008; Schulz et al., 2012). As a result, M1 CST traveling via the posterior edge of the PLIC may have been preferentially lost. Thus, variable function of severely damaged CST from M1 may have rendered its contribution toward recruitment curve properties moot. In such cases, CST from less–damaged territories located anteriorly in higher motor areas could have become recruited due to functional remapping and/or current spreads (Holodny et al., 2005; Ino et al., 2007; Park et al., 2008).

The finding that recruitment curve properties were associated with CST integrity from SMA and PMC can also be understood when considering differences in TMS intensities. Since the ipsilesional hemisphere required a significantly higher intensity to acquire recruitment curves, it is possible that diffuse electric field spreads recruited greater degree of CST from ipsilesional than contralesional higher motor territories (Gerschlager et al., 2001; Teitti et al., 2008). For example, Gerschlager et al. have suggested that stimulation intensities used to induce common neurophysiological metrics from the motor cortex, e.g., MEPs, can activate low threshold premotor pathways as a result of current spreading. Specifically, since the dorsal premotor cortex is located more superficially on the surface of the precentral gyrus in comparison to M1, neurons within these regions are much more likely to have a lower threshold than neuronal components deeper within the central sulcus (M1; Geyer et al., 1996; Klöppel et al., 2008). Thus, even at relatively low RMT, neuronal ensembles from premotor areas may have been preferentially activated in our model.

Regardless of other influences, however, we submit that our observed findings were likely the main result of neurophysiological phenomenon in patients with chronic stroke. Indeed, because we found that the hotspot used for TMS was similar between both the ipsilesional and contralesional hemisphere in patients and between patients and controls (*p* < 0.05 for all comparisons), current spreads may not have contributed as fully. Specifically, given that the distance of current spreads is <15 mm but that the distance from M1 to the PMC is ~25 mm (Rizzo et al., 2004; Boros et al., 2008), the major contributions were likely due to re-mapping of higher motor areas in response to the commonly damaged M1.

### Alterations in recruitment curve properties in the contralesional hemisphere

While we expected that the gain and output of descending tracts would be weaker for the ipsilesional hemisphere, our finding of decreased output in the contralesional hemisphere is surprising (Figure 3). A recent study by Bowden et al has suggested that such a reduced contralesional recruitment curve may reflect limitations in CST function (Bowden et al., 2014). Bowden concluded that recruitment order hierarchy, rather than structural properties, were more affected in the contralesional hemisphere since patients elicited less output from lower threshold fibers than controls, despite comparable MEPs. In contrast, however, we cannot discount that here the contralesional hemisphere presented with reduced CST integrity in certain segments (Figure 4) from both the M1 and PMC. Indeed, reductions in CST integrity in the contralesional hemisphere have been shown to occur as early as 6 months after stroke (Schaechter et al., 2009). Recent work has also suggested that “mirroring damage,” wherein the region homologous to the stroke region on the contralesional side becomes altered, can occur as early as 3 months following a stroke (Granziera et al., 2007, 2010; Crofts et al., 2011). In addition, co-morbidities associated with stroke (e.g., hypertension, diabetes, history of smoking) have been suggested to contribute to small-vessel disease; a condition that could cause subclinical lesions in the contralesional hemisphere (Prins et al., 2005). Thus, since the majority of our patients (*n* = 10) and some of the control subjects (*n* = 3) had small-vessel disease risk factors, this may have also facilitated reduced contralesional integrity. Therefore, taken collectively, along with functional losses, it is conceivable that inherent damage in the contralesional hemisphere either due from the stroke or possible small-vessel disease could have directly impacted measured output.

### Strengths of chosen DTI and TMS metrics

A notable observation was that recruitment curve properties were most related to CST integrity captured along the length of the CST (FA_Mean_ and FA_AUC_). One likely explanation for this finding is based on research inferring that recruitment curve output is influenced by the amount of residual or intact CST function (Table 2; Devanne et al., 1997; Talelli et al., 2006). Thus, only those levels of analysis that accounted for all of the surviving, damaged and degenerated regions within CST, i.e., FA_Mean_ and FA_AUC_, showed significant relationships with recruitment curve metrics. We also observed that analysis of varying regions of CST integrity resulted in significantly different output values. Of note, FA_PLIC_ and FA_Lesion_ were most indicative of damage and only metrics assessing the entire CST (FA_Mean_ and FA_AUC_) were reflective of CST function measured by recruitment curves. Such a result, while explainable given the distinctive definitions of each of the metrics, has implications on future use of DTI metrics in longitudinal studies. Specifically, future researchers should exercise caution when determining how to relate DTI metrics of CST integrity to neurophysiological or functional outcome measures. For example, studies that aim to understand the role of CST damage may benefit from using metrics indicative of regions of most damage (e.g., FA_PLIC_ and FA_Lesion_), while DTI metrics that assess the net integrity of the CST (e.g., FA_Mean_ and FA_AUC_) may be more suitable for studies that evaluate relationships with CST function.

Taken collectively, while our findings here have potential to inform future DTI studies in stroke, they also create enthusiasm for the field of TMS. This is because recruitment curve properties can more closely reflect the graded range of CST damage following stroke in comparison to binary TMS metrics (e.g., RMT, MEP absence/presence; data not shown). Thus, recruitment curves collected using simple, easy-to-administer TMS techniques can closely reflect CST function from areas generally studied with more resource-intensive structural and functional imaging in patients with stroke. For example, physical and occupational therapists could employ the use of recruitment curves to better understand the dynamic and graded changes in CST integrity that occur immediately after stroke in order to maximize a patient's rehabilitation program.

### Limitations

Although we attempted to account for potential problems in our experimental design, our study still suffers from some inherent limitations. First, as a preliminary study, our results only included analysis from 12 patients with chronic stroke, wherein we were unable to record recruitment curves in 3 patients since the severity of their deficit limited our ability to use existing TMS methodology to acquire the curve. Thus, even though our sample size was comparable to sample sizes of other DTI studies in stroke (Ward et al., 2006; Lindberg et al., 2007; Qiu et al., 2011; Allendorfer et al., 2012; Lindenberg et al., 2012; Groisser et al., 2014), future studies with larger sample sizes would be needed to validate the results found here. Similarly, based on methodology from previously conducted work in other groups (Civardi et al., 2001; Butefisch et al., 2003; Rossini and Rossi, 2007), our entire analysis was done in a resting state of the target muscle (FDI). Thus, it is unclear if the relationships noted here can be translated to data collected in an active state (Talelli et al., 2006). Further, we acknowledge that our CST integrity measures encompassed all motor pathways. Thus, any lower extremity deficits or damage to lower extremity motor CST in our patients may have influenced our FA measures, particularly in the SMA. However, while we acknowledge this limitation, we remain optimistic about our findings. This is because our results still emphasize the strength of a more specific modality (TMS) as an ultimate tool to replace structural imaging. We also cannot discount that inclusion of patients with hemorrhagic stroke may have increased data variance; although heterogeneity of lesion size, etiology, and location was similar to reports by other groups (Ward et al., 2006; Lindenberg et al., 2012; Demirtas-Tatlidede et al., 2015). In addition, since comparisons between controls and patients with stroke did not take into account hand dominance, our results may have added confound if patients with stroke displayed a lesion contralateral to their dominant hand. Another limitation in our study is the use of PLIC as the tractography seed. While we chose our seed as to focus on CST controlling voluntary movement (Holodny et al., 2005), future work would need to evaluate the relationship between recruitment curves and tracts descending below the PLIC. Finally, by employing a tractography based analysis, we were unable to relate recruitment curve properties to a specific region of interest that incorporated all descending CST (e.g., non-segmented PLIC). Future work would need to expound upon the results here in order to determine if overall CST FA displayed a similar role in recruitment curve output.

## Conclusions

Our study shows that recruitment curves in patients with chronic stroke may reflect information about CST function mainly from premotor areas but not those from the primary motor cortex. Specifically, we noted that CST integrity from premotor regions was correlated to the output of the recruitment curve (RC AUC_Ipsi/Contra_). This finding is conceivable since higher motor cortices undergo remapping in chronic recovery while M1 CST are substantially damaged (Weiller et al., 1992; Seitz et al., 1998; Bhatt et al., 2007; Ward et al., 2007). Therefore, we suggest caution when interpreting areas that contribute to recovery based on changes in CST function. For example, it may be that even if recruitment curves acquired in the territory of M1 show gains in properties, PMC/SMA located anteriorly could have remapped and instead contributed to recovery.

Another notable finding was that recruitment curve properties were sensitive to integrity along the entire length of CST, taking into account not just the lesion, but also degenerated regions. Based on these results, we suggest that recruitment curves may serve as a viable alternative to time- and cost-intensive imaging modalities when trying to understand CST integrity in a chronic stroke population. We conclude that since different regions of CST damage can uniquely define properties of the recruitment curve, unlike simple TMS metrics that convey binary decisions like recovery or no recovery based on mere presence or absence of MEPs, recruitment curves may serve as a simple, in-expensive means to infer an understanding about damage and degeneration occurring throughout the CST, particularly from re-mapped higher motor regions.

## Author contributions

The initial conception and design of the work was done by KP, DC, AM, AC, and EP. Data was collected and analyzed by KP, NV, SR, VS, CB, and KS. All scripts and analysis of DTI was done by KP and KS. Authors that were in charge of collecting the data were also involved in the interpretation of the data. The primary author of the manuscript was KP. Revisions and critical evaluations, including the addition of substantial intellectual content, were then provided by all authors over the course of several drafts. All authors gave their final approval of the version to be published and are in an agreement to be accountable for any questions related to the accuracy of the work.

### Conflict of interest statement

AM has the following conflicts of interest to disclose: Intelect medical (advisory board, consultant, shareholder), ATI, Enspire and Cardionomics (distribution rights from intellectual property), Functional Neurostimulation (consultant), Deep Brain Innovations (consultant), Medtronic (Fellowship support). The other authors declare that the research was conducted in the absence of any commercial or financial relationships that could be construed as a potential conflict of interest. The reviewer MD and handling Editor declared their shared affiliation, and the handling Editor states that the process nevertheless met the standards of a fair and objective review.

## Abbreviations

TMS: Transcranial Magnetic Stimulation
DWI: Diffusion Weighted Imaging
DTI: Diffusion Tensor Imaging
CST: Corticospinal Tract
M1: Primary Motor Cortex
PMC: Premotor Cortex
SMA: Supplementary Motor Area
MEP: Motor evoked potential.

## References

[B1] Alexander A. L. Lee J. E. Lazar M. Field A. S. (2007). Diffusion tensor imaging of the brain. Neurotherapeutics 4, 316–329. 10.1016/j.nurt.2007.05.011 17599699 PMC2041910

[B2] Allendorfer J. B. Storrs J. M. Szaflarski J. P. (2012). Changes in white matter integrity follow excitatory rTMS treatment of post-stroke aphasia. Restor. Neurol. Neurosci. 30, 103–113. 10.3233/RNN-2011-0627 22233802 PMC3316910

[B3] Bhatt E. Nagpal A. Greer K. H. Grunewald T. K. Steele J. L. Wiemiller J. W.. (2007). Effect of finger tracking combined with electrical stimulation on brain reorganization and hand function in subjects with stroke. Exp. Brain Res. 182, 435–447. 10.1007/s00221-007-1001-5 17562035

[B4] Bogousslavsky J. Regli F. (1990). Anterior cerebral artery territory infarction in the lausanne stroke registry: clinical and etiologic patterns. Arch. Neurol. 47, 144–150. 10.1001/archneur.1990.00530020040012 2302085

[B5] Boroojerdi B. Battaglia F. Muellbacher W. Cohen L. G. (2001). Mechanisms influencing stimulus-response properties of the humancorticospinal system. Clin. Neurophysiol. 112, 931–937. 10.1016/S1388-2457(01)00523-5 11336911

[B6] Boros K. Poreisz C. Münchau A. Paulus W. Nitsche M. A. (2008). Premotor transcranial direct current stimulation (tDCS) affects primary motor excitability in humans. Eur. J. Neurosci. 27, 1292–1300. 10.1111/j.1460-9568.2008.06090.x 18312584

[B7] Boudrias M. H. Belhaj-Saïf A. Park M. C. Cheney P. D. (2006). Contrasting properties of motor output from the supplementary motor area and primary motor cortex in rhesus macaques. Cereb. Cortex. 16, 632–638. 10.1093/cercor/bhj009 16049188

[B8] Bowden J. L. Taylor J. L. McNulty P. A. (2014). Voluntary activation is reduced in both the more- and less-affected upper limbs after unilateral stroke. Front. Neurol. 5:239. 10.3389/fneur.2014.00239 25477862 PMC4237055

[B9] Buffon F. Molko N. Hervé D. Porcher R. Denghien I. Pappata S.. (2005). Longitudinal diffusion changes in cerebral hemispheres after MCA infarcts. J. Cereb. Blood Flow Metab. 25, 641–650. 10.1038/sj.jcbfm.9600054 15689956

[B10] Bütefisch C. M. Netz J. Webling M. Seitz R. J. Hömberg V. (2003). Remote changes in cortical excitability after stroke. Brain 126, 470–481. 10.1093/brain/awg044 12538413

[B11] Calautti C. Jones P. S. Guincestre J. Y. Naccarato M. Sharma N. Day D. J.. (2010). The neural substrates of impaired finger tapping regularity after stroke. Neuroimage 50, 1–6. 10.1016/j.neuroimage.2009.12.012 20004249

[B12] Carroll T. J. Riek S. Carson R. G. (2001). Reliability of the input–output properties of the cortico-spinal pathway obtained from transcranial magnetic and electrical stimulation. J. Neurosci. Methods 112, 193–202. 10.1016/S0165-0270(01)00468-X 11716954

[B13] Carson R. G. Nelson B. D. Buick A. R. Carroll T. J. Kennedy N. C. Cann R. M. (2013). Characterizing changes in the excitability of corticospinal projections to proximal muscles of the upper limb. Brain Stimul. 6, 760–768. 10.1016/j.brs.2013.01.016 23474090

[B14] Chenevert T. L. Brunberg J. A. Pipe J. G. (1990). Anisotropic diffusion in human white matter: demonstration with MR techniques *in vivo*. Radiology 177, 401–405. 10.1148/radiology.177.2.2217776 2217776

[B15] Civardi C. Cantello R. Asselman P. Rothwell J. C. (2001). Transcranial magnetic stimulation can be used to test connections to primary motor areas from frontal and medial cortex in humans. Neuroimage 14, 1444–1453. 10.1006/nimg.2001.0918 11707100

[B16] Crofts J. J. Higham D. J. Bosnell R. Jbabdi S. Matthews P. M. Behrens T. E.. (2011). Network analysis detects changes in the contralesional hemisphere following stroke. Neuroimage 54, 161–169. 10.1016/j.neuroimage.2010.08.032 20728543 PMC3677803

[B17] Cunningham D. A. Machado A. Janini D. Varnerin N. Bonnett C. Yue G.. (2014). The assessment of inter-hemispheric imbalance using imaging and non-invasive brain stimulation in patients with chronic stroke. Arch. Phys. Med. Rehabil. 96 4 Suppl, S94–S103. 10.1016/j.apmr.2014.07.419 25194451 PMC4348350

[B18] Cunningham D. A. Varnerin N. Machado A. Bonnett C. Janini D. Roelle S.. (2015). Stimulation targeting higher motor areas in stroke rehabilitation: A proof-of-concept, randomized, double-blinded placebo-controlled study of effectiveness and underlying mechanisms. Restor. Neurol. Neurosci. 33, 911–926. 10.3233/RNN-150574 26484700 PMC4732280

[B19] Dacosta-Aguayo R. Graña M. Fernández-Andújar M. López-Cáncio E. Caceres C. Bargalló N.. (2014). Structural integrity of the contralesional hemisphere predicts cognitive impairment in ischemic stroke at three months. PLoS ONE 9:e86119. 10.1371/journal.pone.0086119 24475078 PMC3901679

[B20] Dancause N. Barbay S. Frost S. B. Plautz E. J. Chen D. Zoubina E. V.. (2005). Extensive cortical rewiring after brain injury. J. Neurosci. 25, 10167–10179. 10.1523/JNEUROSCI.3256-05.2005 16267224 PMC6725801

[B21] Danielian L. E. Iwata N. K. Thomasson D. M. Floeter M. K. (2010). Reliability of fiber tracking measurements in diffusion tensor imaging for longitudinal study. Neuroimage 49, 1572–1580. 10.1016/j.neuroimage.2009.08.062 19744567 PMC2789889

[B22] Demirtas-Tatlidede A. Alonso-Alonso M. Shetty R. P. Ronen I. Pascual-Leone A. Fregni F. (2015). Long-term effects of contralesional rTMS in severe stroke: safety, cortical excitability, and relationship with transcallosal motor fibers. Neurorehabilitation 36, 51–59. 10.3233/NRE-141191 25547768

[B23] Devanne H. Lavoie B. A. Capaday C. (1997). Input-output properties and gain changes in the human corticospinal pathway. Exp. Brain Res. 114, 329–338. 10.1007/PL00005641 9166922

[B24] Di Lazzaro V. (2004). The physiological basis of transcranial motor cortex stimulation in conscious humans. Clin. Neurophysiol. 115, 255–266. 10.1016/j.clinph.2003.10.009 14744565

[B25] Dum R. P. Strick P. L. (1991). The origin of corticospinal projections from the premotor areas in the frontal lobe. J. Neurosci. 11, 667–689. 1705965 10.1523/JNEUROSCI.11-03-00667.1991 PMC6575356

[B26] Duncan P. W. Propst M. Nelson S. G. (1983). Reliability of the fugl-meyer assessment of sensorimotor recovery following cerebrovascular accident. Phys. Ther. 63, 1606–1610. 6622535 10.1093/ptj/63.10.1606

[B27] Fregni F. Pascual-Leone A. (2007). Technology insight: noninvasive brain stimulation in neurology-perspectives on the therapeutic potential of rTMS and tDCS. Nat. Clin. Pract. Neurol. 3, 383–393. 10.1038/ncpneuro0530 17611487

[B28] Fridman E. A. Hanakawa T. Chung M. Hummel F. Leiguarda R. C. Cohen L. G. (2004). Reorganization of the human ipsilesional premotor cortex after stroke. Brain 127, 747–758. 10.1093/brain/awh082 14749291

[B29] Fries W. Danek A. Scheidtmann K. Hamburger C. (1993). Motor recovery following capsular stroke: Role of descending pathways from multiple motor areas. Brain 116, 369–382. 10.1093/brain/116.2.369 8461971

[B30] Fugl-Meyer A. R. Jääskö L. Leyman I. Olsson S. Steglind S. (1975). The post-stroke hemiplegic patient: I. A method for evaluation of physical performance. Scand. J. Rehab. Med. 7, 13–31. 1135616

[B31] Gerschlager W. Siebner H. R. Rothwell J. (2001). Decreased corticospinal excitability after subthreshold 1 Hz rTMS over lateral premotor cortex. Neurology 57, 449–455. 10.1212/WNL.57.3.449 11502912

[B32] Geyer S. Ledberg A. Schleicher A. Kinomura S. Schormann T. Bürgel U.. (1996). Two different areas within the primary motor cortex of man. Nature 382, 805–807. 10.1038/382805a0 8752272

[B33] Ghosh S. Porter R. (1988). Corticocortical synaptic influences on morphologically identified pyramidal neurones in the motor cortex of the monkey. J. Physiol. 400, 617–629. 10.1113/jphysiol.1988.sp017139 3418539 PMC1191826

[B34] Gladstone D. J. Danells C. J. Black S. E. (2002). The fugl-meyer assessment of motor recovery after stroke: a critical review of its measurement properties. Neurorehabil. Neural Repair 16, 232–240. 10.1177/154596802401105171 12234086

[B35] Grandin C. B. Duprez T. P. Smith A. M. Mataigne F. Peeters A. Oppenheim C.. (2001). Usefulness of magnetic resonance–derived quantitative measurements of cerebral blood flow and volume in prediction of infarct growth in hyperacute stroke. Stroke 32, 1147–1153. 10.1161/01.STR.32.5.1147 11340224

[B36] Granziera C. Ay H. Koniak S. P. Krueger G. Sorensen A. G. (2010). Diffusion tensor imaging shows structural remodeling of stroke mirror region: results from a pilot study. Eur. Neurol. 67, 370–376. 10.1159/000336062 22614706

[B37] Granziera C. D'Arceuil H. Zai L. Magistretti P. J. Sorensen A. G. de Crespigny A. J. (2007). Long-term monitoring of post-stroke plasticity after transient cerebral ischemia in mice using *in vivo* and *ex vivo* diffusion tensor MRI. Open Neuroimag. J. 1, 10–17. 10.2174/1874440000701010010 19018310 PMC2577937

[B38] Groisser B. N. Copen W. A. Singhal A. B. Hirai K. K. Schaechter J. D. (2014). Corticospinal tract diffusion abnormalities early after stroke predict motor outcome. Neurorehabil. Neural Repair. 28, 751–760. 10.1177/1545968314521896 24519021 PMC4128905

[B39] Harris-Love M. L. Morton S. M. Perez M. A. Cohen L. G. (2011). Mechanisms of short-term training-induced reaching improvement in severely hemiparetic stroke patients: a TMS study. Neurorehabil. Neural Repair 25, 398–411. 10.1177/1545968310395600 21343522 PMC3098309

[B40] Henderson R. D. Ridall G. R. Pettitt A. N. McCombe P. A. Daube J. R. (2006). The stimulus-response curve and motor unit variability in normal subjects and subjects with amyotrophic lateral sclerosis. Muscle Nerve 34, 34–43. 10.1002/mus.20561 16634059

[B41] Holodny A. I. Gor D. M. Watts R. Gutin P. H. Ulug A. M. (2005). Diffusion-tensor mr tractography of somatotopic organization of corticospinal tracts in the internal capsule: initial anatomic results in contradistinction to prior reports. Radiology 234, 649–653. 10.1148/radiol.2343032087 15665224

[B42] Hummel F. Celnik P. Giraux P. Floel A. Wu W. H. Gerloff C.. (2005). Effects of non-invasive cortical stimulation on skilled motor function in chronic stroke. Brain 128, 490–499. 10.1093/brain/awh369 15634731

[B43] Ino T. Nakai R. Azuma T. Yamamoto T. Tsutsumi S. Fukuyama H. (2007). Somatotopy of corticospinal tract in the internal capsule shown by functional MRI and di¡usion tensor images. NeuroReport 18, 665–668. 10.1097/WNR.0b013e3280d943e1 17426595

[B44] Jenkinson M. Beckmann C. F. Behrens T. E. Woolrich M. W. Smith S. M. (2012). Fsl. Neuroimage 62, 782–790. 10.1016/j.neuroimage.2011.09.015 21979382

[B45] Johansen-Berg H. Rushworth M. F. Bogdanovic M. D. Kischka U. Wimalaratna S. Matthews P. M. (2002). The role of ipsilateral premotor cortex in hand movement after stroke. Proc. Natl. Acad. Sci. U.S.A. 99, 14518–14523. 10.1073/pnas.222536799 12376621 PMC137915

[B46] Kaelin-Lang A. Cohen L. G. (2000). Enhancing the quality of studies using transcranial magnetic and electrical stimulation with a new computer-controlled system. J. Neurosci. Methods 102, 81–89. 10.1016/S0165-0270(00)00284-3 11000414

[B47] Klöppel S. Baumer T. Kroeger J. Koch M. A. Buchel C. Münchau A.. (2008). The cortical motor threshold reflects microstructural properties of cerebral white matter. Neuroimage 40, 1782–1791. 10.1016/j.neuroimage.2008.01.019 18342540

[B48] Kobayashi S. Hasegawa S. Maki T. Murayama S. (2005). Retrograde degeneration of the corticospinal tract associated with pontine infarction. J. Neurol. Sci. 236, 91–93. 10.1016/j.jns.2005.04.018 15992828

[B49] Kunimatsu A. Aoki S. Masutani Y. Abe O. Hayashi N. Mori H.. (2004). The optimal trackability threshold of fractional anisotropy for diffusion tensor tractography of the corticospinal tract. Magn. Reson. Med. Sci. 3, 11–17. 10.2463/mrms.3.11 16093615

[B50] Lemon R. N. (1999). Neural control of dexterity: what has been achieved? Exp. Brain Res. 128, 6–12. 10.1007/s002210050811 10473734

[B51] Levy R. M. Harvey R. L. Kissela B. M. Winstein C. J. Lutsep H. L. Parrish T. B.. (2016). Epidural electrical stimulation for stroke rehabilitation: results of the prospective, multicenter, randomized, single-blinded everest trial. Neurorehabil. Neural Repair. 30, 107–117. 10.1177/1545968315575613 25748452

[B52] Liang Z. Zeng J. Zhang C. Liu S. Ling X. Xu A.. (2008). Longitudinal investigations on the anterograde and retrograde degeneration in the pyramidal tract following pontine infarction with diffusion tensor imaging. Cerebrovasc. Dis. 25, 209–216. 10.1159/000113858 18216462

[B53] Liepert J. Restemeyer C. Kucinski T. Zittel S. Weiller C. (2005). Motor strokes: the lesion location determines motor excitability changes. Stroke 36, 2648–2653. 10.1161/01.STR.0000189629.10603.02 16269647

[B54] Lindberg P. G. Skejø P. H. Rounis E. Nagy Z. Schmitz C. Wernegren H.. (2007). Wallerian degeneration of the corticofugal tracts in chronic stroke: a pilot study relating diffusion tensor imaging, transcranial magnetic stimulation, and hand function. Neurorehabil. Neural Repair 21, 551–560. 10.1177/1545968307301886 17507645

[B55] Lindenberg R. Zhu L. L. Ruber T. Schlaug G. (2012). Predicting functional motor potential in chronic stroke patients using diffusion tensor imaging. Hum. Brain Mapp. 33, 1040–1051. 10.1002/hbm.21266 21538700 PMC3175010

[B56] Liu Y. Rouiller E. M. (1999). Mechanisms of recovery of dexterity following unilateral lesion of the sensorimotor cortex in adult monkeys. Exp. Brain Res. 128, 149–159. 10.1007/s002210050830 10473753

[B57] Lotze M. Beutling W. Loibl M. Domin M. Platz T. Schminke U.. (2012). Contralesional motor cortex activation depends on ipsilesional corticospinal tract integrity in well-recovered subcortical stroke patients. Neurorehabil. Neural Repair 26, 594–603. 10.1177/1545968311427706 22140195

[B58] Lotze M. Laubis-Herrmann U. Topka H. (2006). Combination of TMS and fMRI reveals a specific pattern of reorganization in M1 in patients after complete spinal cord injury. Restor. Neurol. Neurosci. 24, 97–107. 16720945

[B59] Lowe M. J. Beall E. B. Sakaie K. E. Koenig K. A. Stone L. Marrie R. A.. (2008). Resting state sensorimotor functional connectivity in multiple sclerosis inversely correlates with transcallosal motor pathway transverse diffusivity. Hum. Brain Mapp. 29, 818–827. 10.1002/hbm.20576 18438889 PMC6871176

[B60] Lukács M. Vécsei L. Beniczky S. (2008). Large motor units are selectively affected following a stroke. Clin. Neurophysiol. 119, 2555–2558. 10.1016/j.clinph.2008.08.005 18809353

[B61] MacCabee P. J. Amassian V. E. Eberle L. P. Cracco R. Q. (1993). Magnetic coil stimulation of straight and bent amphibian and mammalian peripheral nerve *in vitro*: locus of excitation. J. Physiol. 460, 201–219. 10.1113/jphysiol.1993.sp019467 8487192 PMC1175209

[B62] Monti R. J. Roy R. R. Edgerton V. R. (2001). Role of motor unit structure in defining function. Muscle Nerve 24, 848–866. 10.1002/mus.1083 11410913

[B63] Neggers S. F. Langerak T. R. Schutter D. J. Mandl R. C. Ramsey N. F. Lemmens P. J.. (2004). A stereotactic method for image-guided transcranial magnetic stimulation validated with fMRI and motor-evoked potentials. Neuroimage 21, 1805–1817. 10.1016/j.neuroimage.2003.12.006 15050601

[B64] Nitsche M. A. Cohen L. G. Wassermann E. M. Priori A. Lang N. Antal A.. (2008). Transcranial direct current stimulation: state of the art 2008. Brain Stimul. 1, 206–223. 10.1016/j.brs.2008.06.004 20633386

[B65] Oldfield R. C. (1971). The assessment and analysis of handedness: the edinburgh inventory. Neuropsychologia 9, 97–113. 10.1016/0028-3932(71)90067-4 5146491

[B66] Park C. H. Kou N. Boudrias M. H. Playford E. D. Ward N. S. (2013). Assessing a standardised approach to measuring corticospinal integrity after stroke with DTI. Neuroimage Clin. 2, 521–533. 10.1016/j.nicl.2013.04.002 24179804 PMC3777681

[B67] Park J. K. Kim B. S. Choi G. Kim S. H. Choi J. C. Khang H. (2008). Evaluation of the somatotopic organization of corticospinal tracts in the internal capsule and cerebral peduncle: results of diffusion-tensor MR tractography. Korean J. Radiol. 9, 191–195. 10.3348/kjr.2008.9.3.191 18525220 PMC2627255

[B68] Plow E. B. Cunningham D. A. Varnerin N. Machado A. (2014). *Rethinking stimulation of the brain in stroke* rehabilitation: why higher motor areas might be better alternatives for patients with greater impairments. Neuroscientist. 10.1177/1073858414537381 24951091 PMC4440790

[B69] Prins N. D. van Dijk E. J. den Heijer T. Vermeer S. E. Jolles J. Koudstaal P. J.. (2005). Cerebral small-vessel disease and decline in information processing speed, executive function and memory. Brain 128, 2034–2041. 10.1093/brain/awh553 15947059

[B70] Puig J. Pedraza S. Blasco G. Daunis I. E. J. Prados F. Remollo S.. (2011). Acute damage to the posterior limb of the internal capsule on diffusion tensor tractography as an early imaging predictor of motor outcome after stroke. AJNR Am. J. Neuroradiol. 32, 857–863. 10.3174/ajnr.A2400 21474629 PMC7965569

[B71] Qiu M. Darling W. G. Morecraft R. J. Ni C. C. Rajendra J. Butler A. J. (2011). White matter integrity is a stronger predictor of motor function than BOLD response in patients with stroke. Neurorehabil. Neural Repair 25, 275–284. 10.1177/1545968310389183 21357529 PMC3579586

[B72] Ridding M. C. Rothwell J. C. (1997). Stimulus/response curves as a method of measuring motor cortical excitability in man. Electrocephalogr. Clin. Neurophysiol. 105, 340–344. 10.1016/S0924-980X(97)00041-6 9362997

[B73] Rizzo V. Siebner H. R. Modügno N. Pesenti A. Munchau A. Gerschlager W.. (2004). Shaping the excitability of human motor cortex with premotor rTMS. J. Physiol. 554, 483–495. 10.1113/jphysiol.2003.048777 14555728 PMC1664763

[B74] Rossi S. Hallett M. Rossini P. M. Pascual-Leone A. Safety of TMS Consensus Group. (2009). Safety, ethical considerations, and application guidelines for the use of transcranial magnetic stimulation in clinical practice and research. Clin. Neurophysiol. 120, 2008–2039. 10.1016/j.clinph.2009.08.016 19833552 PMC3260536

[B75] Rossini P. M. Rossi S. (2007). Transcranial magnetic stimulation: diagnostic, therapeutic, and research potential. Neurology 68, 484–488. 10.1212/01.wnl.0000250268.13789.b2 17296913

[B76] Sakaie K. E. Lowe M. J. (2007). An objective method for regularization of fiber orientation distributions derived from diffusion-weighted MRI. Neuroimage 34, 169–176. 10.1016/j.neuroimage.2006.08.034 17030125

[B77] Schaechter J. D. Fricker Z. P. Perdue K. L. Helmer K. G. Vangel M. G. Greve D. N.. (2009). Microstructural status of ipsilesional and contralesional corticospinal tract correlates with motor skill in chronic stroke patients. Hum. Brain Mapp. 30, 3461–3474. 10.1002/hbm.20770 19370766 PMC2780023

[B78] Schulz R. Park C. H. Boudrias M. H. Gerloff C. Hummel F. C. Ward N. S. (2012). Assessing the integrity of corticospinal pathways from primary and secondary cortical motor areas after stroke. Stroke 43, 2248–2251. 10.1161/STROKEAHA.112.662619 22764214 PMC3477824

[B79] Seitz R. J. Höflich P. Binkofski F. Tellmann L. Herzog H. Freund H.-J. (1998). Role of the premotor cortex in recovery from middle cerebral artery infarction. Arch. Neurol. 55, 1081–1088. 10.1001/archneur.55.8.1081 9708958

[B80] Shellock F. G. (2014). Available online at: www.MRIsafety.com

[B81] Sidaros A. Engberg A. W. Sidaros K. Liptrot M. G. Herning M. Petersen P.. (2008). Diffusion tensor imaging during recovery from severe traumatic brain injury and relation to clinical outcome: a longitudinal study. Brain 131, 559–572. 10.1093/brain/awm294 18083753

[B82] Soares J. M. Marques P. Alves V. Sousa N. (2013). A hitchhiker's guide to diffusion tensor imaging. Front. Neurosci. 7:31. 10.3389/fnins.2013.00031 23486659 PMC3594764

[B83] Stinear C. M. Barber P. A. Petoe M. Anwar S. Byblow W. D. (2012). The PREP algorithm predicts potential for upper limb recovery after stroke. Brain 135, 2527–2535. 10.1093/brain/aws146 22689909

[B84] Stinear C. M. Barber P. A. Smale P. R. Coxon J. P. Fleming M. K. Byblow W. D. (2007). Functional potential in chronic stroke patients depends on corticospinal tract integrity. Brain 130, 170–180. 10.1093/brain/awl333 17148468

[B85] Takeuchi N. Tada T. Chuma T. Matsuo Y. Ikoma K. (2007). Disinhibition of the premotor cortex contributes to a maladaptive change in the affected hand after stroke. Stroke 38, 1551–1556. 10.1161/STROKEAHA.106.470187 17363726

[B86] Talelli P. Greenwood R. J. Rothwell J. C. (2006). Arm function after stroke: neurophysiological correlates and recovery mechanisms assessed by transcranial magnetic stimulation. Clin. Neurophysiol. 117, 1641–1659. 10.1016/j.clinph.2006.01.016 16595189

[B87] Talelli P. Ewas A. Waddingham W. Rothwell J. C. Ward N. S. (2008). Neural correlates of age-related changes in cortical neurophysiology. NeuroImage 40, 1772–1781. 10.1016/j.neuroimage.2008.01.039 18329904 PMC3715371

[B88] Teitti S. Maatta S. Säisänen L. Könönen M. Vanninen R. Hannula H.. (2008). Non-primary motor areas in the human frontal lobe are connected directly to hand muscles. Neuroimage 40, 1243–1250. 10.1016/j.neuroimage.2007.12.065 18289883

[B89] Thickbroom G. W. Byrnes M. L. Archer S. A. Mastaglia F. L. (2002). Motor outcome after subcortical stroke: MEPs correlate with hand strength but not dexterity. Clin. Neurophysiol. 113, 2025–2029. 10.1016/S1388-2457(02)00318-8 12464343

[B90] Ward N. (2011). Assessment of cortical reorganisation for hand function after stroke. J. Physiol. 589, 5625–5632. 10.1113/jphysiol.2011.220939 22063630 PMC3249038

[B91] Ward N. S. Newton J. M. Swayne O. B. Lee L. Frackowiak R. S. Thompson A. J.. (2007). The relationship between brain activity and peak grip force is modulated by corticospinal system integrity after subcortical stroke. Eur. J. Neurosci. 25, 1865–1873. 10.1111/j.1460-9568.2007.05434.x 17432972 PMC3715370

[B92] Ward N. S. Newton J. M. Swayne O. B. Lee L. Thompson A. J. Greenwood R. J.. (2006). Motor system activation after subcortical stroke depends on corticospinal system integrity. Brain 129, 809–819. 10.1093/brain/awl002 16421171 PMC3717515

[B93] Weiller C. Chollet F. Friston K. J. Wise R. J. S. Frackowiak R. S. J. (1992). Functional Reorganization of the brain in recovery from striatocapsular idarction in man. Ann. Neurol. 31, 463–472. 10.1002/ana.410310502 1596081

[B94] Wittenberg G. F. Schaechter J. D. (2009). The neural basis of constraint-induced movement therapy. Curr. Opin. Neurol. 22, 582–588. 10.1097/WCO.0b013e3283320229 19741529

[B95] Zeiler S. R. Gibson E. M. Hoesch R. E. Li M. Y. Worley P. F. O'Brien R. J.. (2013). Medial premotor cortex shows a reduction in inhibitory markers and mediates recovery in a mouse model of focal stroke. Stroke 44, 483–489. 10.1161/STROKEAHA.112.676940 23321442 PMC4086919

[B96] Zhang M. Sakaie K. E. Jones S. E. (2013). Logical foundations and fast implementation of probabilistic tractography. IEEE Trans. Med. Imag. 32, 1397–1410. 10.1109/TMI.2013.2257179 23568498

[B97] Zhu L. L. Lindenberg R. Alexander M. P. Schlaug G. (2010). Lesion load of the corticospinal tract predicts motor impairment in chronic stroke. Stroke 41, 910–915. 10.1161/STROKEAHA.109.577023 20378864 PMC2886713

